# Large-Scale Analysis Exploring Evolution of Catalytic Machineries and Mechanisms in Enzyme Superfamilies

**DOI:** 10.1016/j.jmb.2015.11.010

**Published:** 2016-01-29

**Authors:** Nicholas Furnham, Natalie L. Dawson, Syed A. Rahman, Janet M. Thornton, Christine A. Orengo

**Affiliations:** 1 Department of Infectious and Tropical Diseases, London School of Hygiene and Tropical Medicine, London WC1E 7HT, United Kingdom; 2 Institute of Structural and Molecular Biology, University College London, Darwin Building, Gower Street, London WC1E 6BT, United Kingdom; 3 European Molecular Biology Laboratory, European Bioinformatics Institute, Wellcome Trust Genome Campus, Hinxton, Cambridge CB10 1SD, United Kingdom

**Keywords:** EC, Enzyme Commission, IUBMB, International Union of Biochemistry and Molecular Biology, CSA, Catalytic Site Atlas, SSG, structurally similar groups, TIM, triosephosphate isomerase, CMSS, Catalytic Machinery Similarity Score, NP, normalised proportion, GOX, glycolate oxidase, FCB, flavocytochrome *b*2, FMN, flavin mononucleotide, protein domain evolution, enzyme function evolution, catalytic residues, protein domain superfamilies, enzyme reaction chemistry

## Abstract

Enzymes, as biological catalysts, form the basis of all forms of life. How these proteins have evolved their functions remains a fundamental question in biology. Over 100 years of detailed biochemistry studies, combined with the large volumes of sequence and protein structural data now available, means that we are able to perform large-scale analyses to address this question. Using a range of computational tools and resources, we have compiled information on all experimentally annotated changes in enzyme function within 379 structurally defined protein domain superfamilies, linking the changes observed in functions during evolution to changes in reaction chemistry. Many superfamilies show changes in function at some level, although one function often dominates one superfamily. We use quantitative measures of changes in reaction chemistry to reveal the various types of chemical changes occurring during evolution and to exemplify these by detailed examples. Additionally, we use structural information of the enzymes active site to examine how different superfamilies have changed their catalytic machinery during evolution. Some superfamilies have changed the reactions they perform without changing catalytic machinery. In others, large changes of enzyme function, in terms of both overall chemistry and substrate specificity, have been brought about by significant changes in catalytic machinery. Interestingly, in some superfamilies, relatives perform similar functions but with different catalytic machineries. This analysis highlights characteristics of functional evolution across a wide range of superfamilies, providing insights that will be useful in predicting the function of uncharacterised sequences and the design of new synthetic enzymes.

## Highlights

• Examining how enzyme function evolves using sequence, structure, and reaction mechanism data.
• Quantifying changes in reaction mechanisms reveals how function has diverged in many superfamilies.
• Homologous domains frequently use different catalytic residues, which sometimes perform the same enzyme chemistry.
• This large-scale analysis has significance in protein function prediction and enzyme design.

## Graphical abstract

**Figure:**
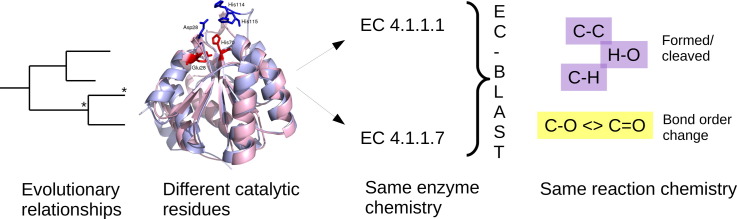

## Introduction

Enzyme-associated protein domains are found in nearly 70% of the superfamilies in the CATH [1] domain family database (1817 out of 2626, CATH version 3.5) and make up approximately 47% (257,522 out of 548,454 protein sequences) of all protein sequences in the reviewed section of the UniProt Knowledgebase (UniProtKB) [2]. Protein domains that are solely responsible for enzyme catalysis however (i.e., that contain the majority of the catalytic residues) are found in approximately 14% (379 out of 2626) CATH superfamilies. The large volume of sequence data now available, for example, the ~ 46 million sequence entries in the 2015_04 release of UniProtKB, combined with the cumulative knowledge of decades of biochemical analysis of enzymes, now make large-scale studies of evolution of enzyme function attractive. Whereas previous studies of enzymes have been limited by the lack of available data to the study of single enzymes or specific enzyme superfamilies, we now have sufficient data to explore the evolutionary relationships through structural, sequence, and functional information for hundreds of enzyme superfamilies.

Functional diversity can arise as a result of selective evolutionary pressures. The duplication of genetic material provides the necessary molecular elements needed to form additional and/or novel functions. Here we consider the divergence of enzyme functions.

Early studies of protein families [3] revealed interesting examples of evolutionary relatives (e.g., mandelate racemase and muconate lactonising enzyme) catalysing different chemical reactions. Novel functions often evolve through incremental residue mutations, which can lead to differences in the catalytic machinery of an active site. Other mechanisms modifying protein functions during evolution include the following: the insertion or deletion of residues (indels), generally occurring in the loop regions between core secondary structure elements [4]; oligomerisation, where two or more copies of the same protein, or at least one copy of two or more different proteins, form a protein complex; gene fusion; gene fission; alternative gene splicing; exon shuffling through intronic recombination; and post-translational modifications [5]. The divergence of function can also be a product of differences in the metal ions, as well as other co-factors, present in an active site.

Large-scale studies of the chemistries performed by different relatives in enzyme superfamilies (e.g., Todd *et al.* [4]) have shown that, whilst there can be considerable diversity in substrate specificity, the reaction chemistry is usually retained where “chemistry” refers to the mechanism of changing substrate into product and includes the nature of the intermediates. Babbit and Gerlt analysed four enzyme superfamilies (the Enolase, *N*-Acetylneuraminate Lyase, Crotonase, and Vicinal Oxygen Chelate superfamilies) in which relatives share the same structural fold, or scaffold, but can catalyse different catalytic reactions [6]. In each, they found conservation of catalytic groups needed to catalyse the partial chemical reaction common to all superfamily members. There was also evidence of new catalytic groups, recruited to the active site, leading to the evolution of new catalytic activities.

Enzyme evolution has also been explored from the perspective of metabolic pathways. Teichmann *et al.* found that, as the number of domains within a family increased, the number of pathways in which the family was involved also increased, that is, suggesting recruitment of relatives for the particular function they bring to a new pathway [7]. Further studies on small molecule metabolic pathways [8] found additional support (from phylogenetic, metabolic, and structural analyses) for evolution of pathways through a chemistry-driven “patchwork” model [9], again favouring conservation of enzyme chemistry.

However, more recent studies exploiting the much larger volumes of data currently available have shown occurrences of considerable variation in enzyme chemistry within some superfamilies. Furnham *et al.* derived phylogenetic trees for CATH enzyme superfamilies [10] to explore evolution of functions using a new resource, FunTree, which links these evolutionary data to data on substrates/products and reaction chemistries from MACiE (Mechanism, Annotation, and Classification in Enzymes) [11] and catalytic residue data from the Catalytic Site Atlas (CSA) [12].

It is also important to consider the role that promiscuity may play in functional divergence. Khersonsky and Tawfik review several studies highlighting the fact that duplicated proteins can be recruited to carry out different functions without any changes at the DNA level [13], that is, moonlighting proteins. Alternatively, a new protein function can evolve through promiscuous intermediates, and it is these intermediates that are modified following gene duplication. A recent large-scale study by Huang *et al.* [44] tested for promiscuous enzyme activity in members of the haloalkanoic acid dehalogenase superfamily and found that most had the ability to react with at least five substrates. These promiscuous activities make function prediction challenging, as highlighted in Mashiyama *et al.* [14] where the many sub-groups of the Cytosolic Glutathione Transferase superfamily are shown to catalyse reactions that widely overlap with other sub-groups and to be highly promiscuous. Baier and Tokuriki examined promiscuity in the metallo-beta-lactamase superfamily [15]. They found that members catalysed 1.5 reactions on average. These metallo-beta-lactamase enzymes play the same catalytic role and the presence of two Zn^2 +^ ions within the shared binuclear active-site centre is reported to be an important feature for native and promiscuous activities. It is thought that the plasticity of the metal ions helps us to introduce promiscuous activities.

An interesting question in the context of understanding evolutionary changes and designing novel enzyme functions is the extent to which residues in the active site have changed during evolution and the effects on the substrates bound and the chemistry performed by the relative.

The active site of an enzyme is typically found in a large pocket on the protein surface [16], which allows a ligand substrate to bind in a solvent-free environment. The increasing amount of active site and catalytic residue data, available in public resources (e.g., the CSA [12], Inferred Biomolecular Interactions Server [17], firestar [18]), has enabled large-scale studies on the location of catalytic sites. As regards the arrangements of catalytic residues in active sites, many studies have shown that although catalytic residues tend to be conserved in their structural location, they are not necessarily conserved in sequence. Furthermore, on a dataset of 31 superfamilies analysed, Todd *et al.* reported that over one-third showed some variation in their catalytic machineries [4]. Whilst some homologues use the same catalytic machinery to catalyse a variety of enzymatic reactions, others use different catalytic machinery to catalyse very similar reactions [19].

Information on enzyme families has expanded significantly over the last 10 years, making it timely to revisit the data and seek further insights on protein function evolution and how changes in the catalytic machinery in protein families impact the chemistries performed by different relatives. We reconstruct the evolutionary histories of 379 CATH superfamilies, using existing enzyme data to predict ancestral enzyme function. Changes in catalytic machineries between CATH functional sub-families within superfamilies are explored and we also investigate whether a change in catalytic machinery directly links to changes in enzyme chemistry using reaction chemistry similarity measures.

## Results and Discussion

### Cataloguing changes in function during evolution

**Fig. 1:**
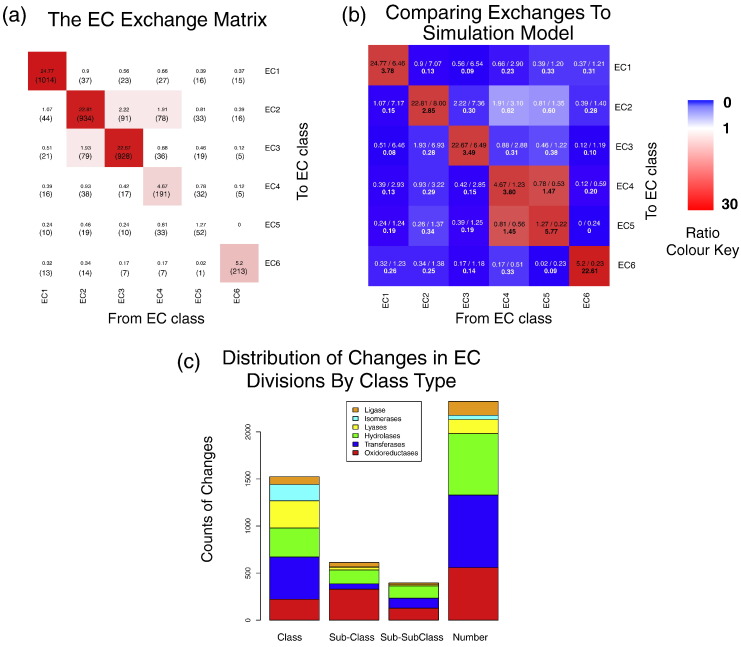
Changes in enzyme function by EC number classification across 379 domain superfamilies. (a) A matrix counting the number of changes from one EC number to another based on the phylogenetic tree generated in FunTree. The changes from one function to another are based on node maximum-likelihood ancestral character estimation and leaf annotations. The counts, which are expressed as a percentage of the total number of observed changes, are shown in each cell and define the cell colouring, based on a red intensity scale. (b) Comparing observed changes in function to a model of random changes. Each cell in the matrix reports the ratio of the number of observed changes as a percentage (from a) to the number of changes simulated by randomly selecting two EC numbers based on the EC numbers catalogued in FunTree. The ratio is shown in boldface, with the actual percentages shown just above. The matrix is coloured based on the ratio, where under-representation of changes is shown on a blue-to-white scale, changes matching the expected random model are shown in white, and over-represented changes are shown on a white-to-red scale. (c) The number changes between EC numbers showing the differences between the divisions in the EC classification. Each class is shown by a unique colour.

The major goal of this research was to discover changes in (enzyme) function within a superfamily and to examine from a structural perspective how these changes came about. The results of our analysis of 379 domain superfamilies are presented in an Enzyme Commission (EC) exchange matrix (Fig. 1a). Consistent with previous observations [10], most changes of function occur within EC classes and the number of changes within each class is approximately proportional to the number of divisions within the class. Some exchanges do occur between different EC classes (e.g., from oxidoreductase to transferase) although there are very few changes between the ligases (EC 6) and the other classes. Since we can provide directionality to the changes by estimating the ancestral function, we observe that the exchange matrix is non-symmetrical. Some inter-class changes appear to be more common in one direction than the other; for example, changes from transferases to isomerases (0.81%—the percentage of changes in the total number of observed changes across and within all classes) are more common than from isomerases to transferases (0.46%), though the numbers of changes are small.

To ascertain if there were significant under- or over-representation of certain changes, we compared the matrix of observed changes to an expectation model that simulates the chances of one function changing to another based on the EC numbers catalogued in FunTree, which also intrinsically takes into account the bias introduced by the sizeable differences in the granularity of the classification system. The ratio of the observed over expected changes is calculated (see Fig. 1b). This shows that there is significant over-representation of changes within-class; that is, an oxidoreductase preferentially evolves to another oxidoreductase, albeit in a different sub-class or sub-subclass. Overall, 81.4% of changes occur within an EC class, compared to just 22.6% that would be expected based on a random model.

The paucity of inter-class changes suggests that changing overall chemistry is more challenging than changing substrates during evolution. Perhaps it requires many complementary mutations to occur, each of which does not disrupt the enzyme's activity to the point of being deleterious to the fitness of the organism. Alternatively, only a very few residues might be candidates for changing the chemistry, whereas many could change the binding. Also, the order in which mutations occur might be critical [20], [21], [22]. Previous hierarchical clustering of the six primary EC classes [23], based only on bond order changes, showed that the oxidoreductases, lyases, and isomerases cluster together (EC classes 1, 4, and 5) as do the transferases, hydrolases, and ligases (EC classes 2, 3, and 6). However, the observed evolutionary changes in function, as seen in the EC exchange matrix, do not reflect this clustering, on average, showing no particular preference for exchanges within these clusters. The one exception to within-class exchanges is that isomerases (EC 5) and lyases (EC 4) inter-convert frequently (see Fig. 1b). Intra-class changes are mostly at the fourth level (serial number) of the EC number and are broadly proportional to the number of class divisions (see Fig. 1c). Most sub-class changes occur within the oxidoreductases, where almost one-half of the superfamilies show this behaviour. On the other hand, changes in sub-class level in the ligases are confined to just 18% of ligase superfamilies and most of the changes are restricted to just two superfamilies both ATP-Grasp folds [CATH IDs 3.30.1490.20 (acetyl-CoA carboxylase activity ATP-like) and 3.30.470.20 (carbamoyl-phosphate synthase activity-like)]. In fact, recent analysis [24] has suggested that these two superfamilies are related, which indicates that the numbers of sub-class changes are confined to an even smaller proportion of superfamilies. Very few sub-class (second level of the EC classification) and sub-subclass (third level of the EC classification) changes occur in the lyases and isomerases, reflecting the nature of the EC classification.

### What proportion of enzyme functions have arisen from another function

We observe that 2994 unique enzymes [i.e., considering unique Enzyme Classification numbers (EC 4)] fall into 379 CATH domain superfamilies accounting for ~ 56% (97,116 out of 173,536) of all domain sequences in CATH. This suggests that 2615 enzyme functions (i.e., 2994 − 379) have evolved from another function or from an ancestor with generic functionality, comprising over 87% of all functions. Furthermore, since 1779 of the 2994 EC 4 (59%) are associated with at least two superfamilies, this suggests that more than half of the enzyme functions have arisen in more than one superfamily during evolution. A caveat in this analysis is that some superfamilies in a given fold may be homologous. Prior to each CATH release, we perform HMM–HMM profile comparisons between all superfamily pairs to merge any closely related superfamilies. However, it could be that some close relationships are missed and therefore functions that apparently emerged in two different superfamilies could have in fact emerged in the same superfamily, and our values are over-estimated. Further analysis has shown that 585 of the 1779 EC 4 (~ 33%) associated with more than two superfamilies are from superfamilies within the same fold group.

### Analysing changes in function by comparing reactions

**Fig. 2:**
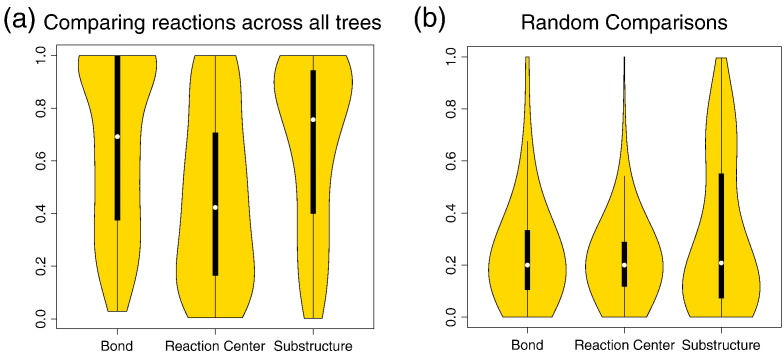
Summary of reaction similarities for all changes in enzyme function across 379 domain superfamilies. Violin plots showing the distribution of bond order, reaction centre, and sub-structure similarity scores for changes between two functions based on (a) using the changes in reactions found within the FunTree trees and (b) using the functions found in FunTree with each pair of reaction being randomly selected iterated 5000 times.

Though the EC numbering system is good for curating enzyme functions, it is not useful for making quantitative comparisons between reaction chemistries. By using EC-BLAST, we can quantify the functional changes observed in the EC exchange matrix by calculating similarities in bond order changes, reaction centres, and substrate/product sub-structures (Fig. 2). The figure shows two sets of distributions, representing scores for all observed changes in function (Fig. 2a) and the random distributions (Fig. 2b) against which the others may be compared. As expected for all three measures, the observed comparison scores are much larger (i.e., changes are smaller and scores closer to 1.0) than the random comparisons. Comparison of the different scores shows that bond changes and sub-structure comparison scores show much less difference between functions than the reaction centre scores, indicating that the local environment around the bonds that are cleaved may be quite different with the presence of different chemical groups. These observations apply equally to all enzyme classes (Fig. S1, Fig. S2, Fig. S3, Fig. S4, Fig. S5, Fig. S6, Fig. S7, Fig. S8, Fig. S9).

### Inspection of specific superfamilies

**Fig. 3:**
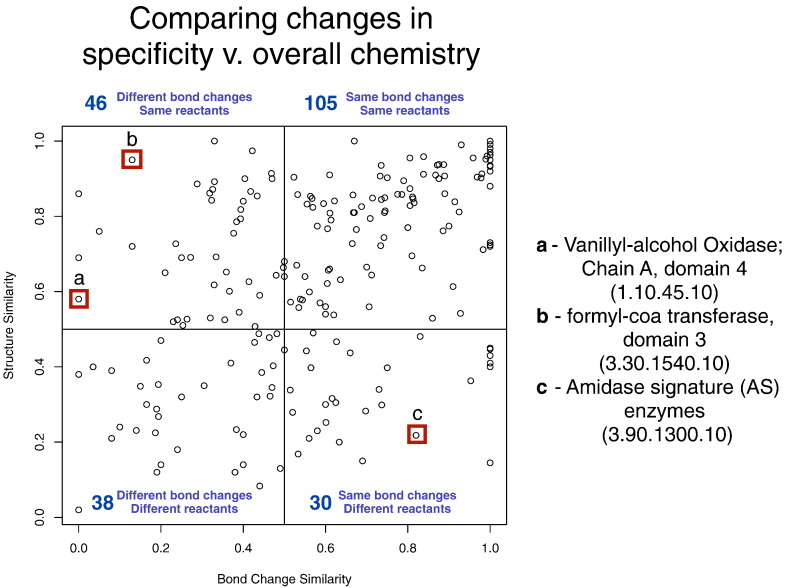
Comparing changes in specificity compared to overall reaction chemistry. A red box highlights example superfamilies, listed on the right and discussed in the main text. The plot is dived into four sections broadly representing the four possible types of changes, with the total number of superfamilies in each quadrant given in blue. Not all superfamilies are present as some are mono-functional, whilst others contain reactions that are unbalanced (i.e., have a different number of atoms on one side of the reaction compared to the other, often from the inclusion of R groups) and thus not amenable to the EC-BLAST method. A summary of the superfamilies used in this analysis is provided in Table S22.

To gain an overview of how different superfamilies are changing chemistry *with* changing substrates, we compared the average bond similarity score to the average sub-structure similarity score as calculated by EC-BLAST for each superfamily (Fig. 3). Each point on the plot represents the average score values of all the exchanges observed in a given superfamily. As expected, most families (top-right quadrant) show a conservative evolution, with only small changes in bond order and structure of the reactants across relatives. Others though demonstrate a much greater diversity in either reaction chemistry or substrates or both.

**Fig. 4:**
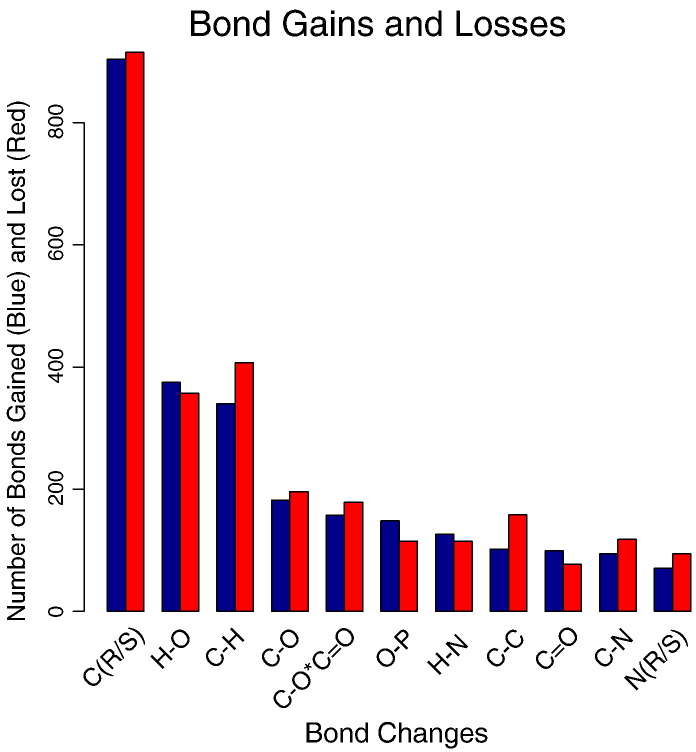
Summary of top 10 bond types that are gained and lost through the evolution of enzyme function within 379 domain superfamilies. For each reaction, the type of bond changes can be determined. Where a change in function in observed within a tree, we determine the difference in the number and type of bond changes between the two reactions. Where a bond is present in the ancestral function and not in the more modern function, this is counted as a bond gain and vice versa for a loss. These are then summated for all superfamilies in FunTree, with the top 10 bond gains (blue) shown with their losses (red).

Below, we highlight a few specific examples to illustrate the different paradigms we observe:

(i) Similar reactants with very different chemistry (Fig. 4a and b)
  A superfamily that has similar reactants but different bond changes is the Vanillyl-Alcohol Oxidase superfamily (CATH ID 1.10.45.10) (Fig. S10). The function evolves from vanillyl-alcohol oxidase (EC 1.1.3.38), in which three OH bonds and one CH bond are changed plus a change from a double to a single O–O bond to 4-methylphenol dehydrogenase (EC 1.17.99.2), in which one C–O bond is formed plus an R/S change in stereochemistry. The bond changes are therefore completely different. However, the reactants are rather similar, both including a six-membered carbon ring. Both reactions involve a common flavin adenine dinucleotide co-factor, with common steps in the enzyme mechanisms, but although both involve a histidine, this residue is not equivalent in the two proteins and the difference in chemistry occurs due to other residues recruited within the active-site cleft.
  A superfamily that has similar reactants but different bond changes is the Vanillyl-Alcohol Oxidase superfamily (CATH ID 1.10.45.10) (Fig. S10). The function evolves from vanillyl-alcohol oxidase (EC 1.1.3.38), in which three OH bonds and one CH bond are changed plus a change from a double to a single O–O bond to 4-methylphenol dehydrogenase (EC 1.17.99.2), in which one C–O bond is formed plus an R/S change in stereochemistry. The bond changes are therefore completely different. However, the reactants are rather similar, both including a six-membered carbon ring. Both reactions involve a common flavin adenine dinucleotide co-factor, with common steps in the enzyme mechanisms, but although both involve a histidine, this residue is not equivalent in the two proteins and the difference in chemistry occurs due to other residues recruited within the active-site cleft.
(ii) Conserved bond order changes with very different reactants (Fig. 4c)
  In contrast, in the Amidase Signature enzyme superfamily (CATH ID 3.90.1300.10), we observe a change from amidase (EC 3.5.1.4) to 6-aminohexanoate-cyclic-dimer hydrolase (EC 3.5.2.12) (Fig. S11). Both reactions share almost the same bond changes (with NH, OH, and CO bond changes in common) but the reactants involved are very different. Although five of the seven catalytic residues are conserved, they are mostly located in loop regions, which presumably allow the active site to accommodate very different reactants, yet they perform the same chemistry.
  In contrast, in the Amidase Signature enzyme superfamily (CATH ID 3.90.1300.10), we observe a change from amidase (EC 3.5.1.4) to 6-aminohexanoate-cyclic-dimer hydrolase (EC 3.5.2.12) (Fig. S11). Both reactions share almost the same bond changes (with NH, OH, and CO bond changes in common) but the reactants involved are very different. Although five of the seven catalytic residues are conserved, they are mostly located in loop regions, which presumably allow the active site to accommodate very different reactants, yet they perform the same chemistry.

### Changing bond types in evolution

In automatically comparing the bond changes between two reactions, it is possible to catalogue the bond types that have been gained and those that are lost. Again using the phylogenetic trees and ancestral function estimation, it is possible to transverse the trees summarising across all superfamilies the changes in gain/loss of different bond types (Fig. 4 and Fig. S12). The prevalence of the types of bonds being altered is in keeping with the prevalence of these bonds in all International Union of Biochemistry and Molecular Biology (IUBMB) reactions from previous analysis based on all-by-all comparison on IUBMB reactions [23], though the ordering within the top 10 is different.

By far the most prevalent change in bond type is a change in chirality at a carbon chiral centre [C(R/S)], followed by the making or breaking of an H–O or a C–H bond. The most conserved bond change in two related reactions is the O–H bond change (Fig. S12). The most striking feature of this analysis is the overall neutrality in the gain and loss of any bond type.

The sequences and their functional annotations used in this analysis come from the reviewed section of UniProtKB with varying degrees of explicit experimental characterisation. Only a relatively small proportion has been experimentally validated. However, as part of the manual curation process, functional annotations are corroborated with the existing literature. Some annotations made by means of similarity to existing experimentally validated homologues may be incorrect or only partially correct [25] but the fact that they have been manually checked in the literature makes us more confident in them. In addition, there are many sequences, excluded from this analysis, that have yet to be either curated or experimentally characterised. New tools such as EC-BLAST can help recognise whether a new uncharacterised protein has a similar function to something already known and, if not, is a novel function that needs to be experimentally characterised. Undoubtedly, further analysis of the evolution of new functions will benefit hugely from researchers experimentally exploring and characterising enzymes with unknown function.

### Changes in catalytic machinery between relatives

Ideally, to understand how different functions evolve, catalytic mechanisms should be compared, but unfortunately, these are not well defined for many enzymes. However, we do know which residues in a binding site are conserved and involved in catalysis; thus, here we compare those catalytic residues and explore how they have changed during evolution. Information on catalytic residues was extracted from the CSA database for each CATH functional family, including known catalytic residues and other residues implicated in the chemistry, for example, stabilising an intermediate state. Comparisons of these residues within superfamilies, especially where the function has changed, revealed active-site diversity across a superfamily and determined to what extent changes in catalytic residues are associated with changes in chemistry and/or substrate specificity.

A total of 101 (out of the 379) CATH enzyme superfamilies have at least two families with different functions in which one or more of the domains have literature-based catalytic residue annotations. Catalytic residues were compared in terms of both physiochemical similarity and equivalence in the sequence alignment or in three dimensions.

**Fig. S13:**
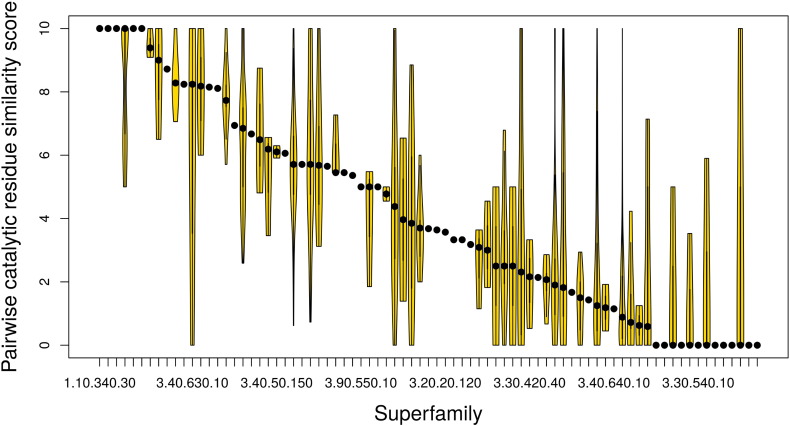
The range of catalytic residue similarity between pairs of functional families in each superfamily. Violin plots show the variation in catalytic residue similarity between pairs of functional families in a given superfamily using the partially annotated approach and the structure-based sequence alignment-based protocol. Each violin represents one superfamily.

**Fig. 5:**
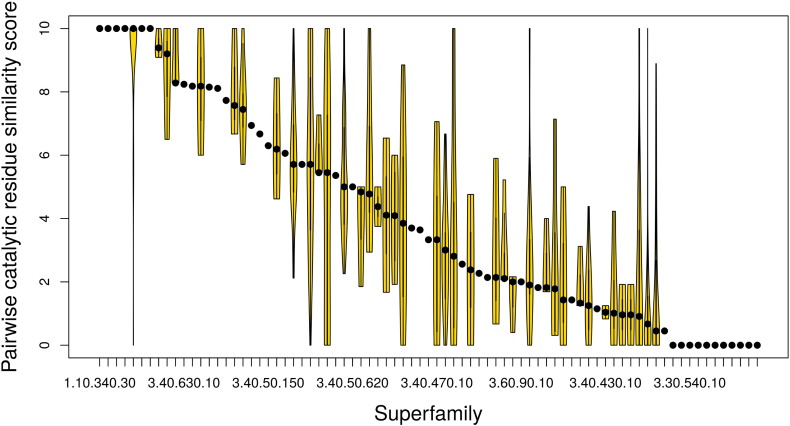
The range of catalytic residue similarity between pairs of functional families in each enzyme superfamily. Violin plots show the variation in catalytic residue similarity between pairs of functional families in a given superfamily using the partially annotated approach and the structure-based, sequence-alignment-based protocol. Each violin represents one superfamily.

Figure 5 shows average values of similarity in catalytic residues between pairs of functional families [Catalytic Machinery Similarity Score (CMSS)], together with the range of pairwise similarities observed, for each superfamily studied. There is an almost continuous distribution of similarity, from complete conservation in some superfamilies to zero similarity between catalytic residues in others using the structure-based sequence alignment protocol (Fig. 5). Similar results are obtained from three-dimensional superpositions, which compare co-located (superimposed) catalytic residues, regardless of their positions in the sequence (see Fig. S13). We observe that, during evolution, catalytic residues change both in their physicochemical characteristics and in their locations within the active site. We also observe that the annotations in the CSA often omit catalytic residues in one family that have been implicated in another family (even when they are present and co-located in the structures). This reflects the challenge of identifying “catalytic residues”, with different authors in the literature using different criteria in describing residues as “catalytic”.

**Fig. S14:**
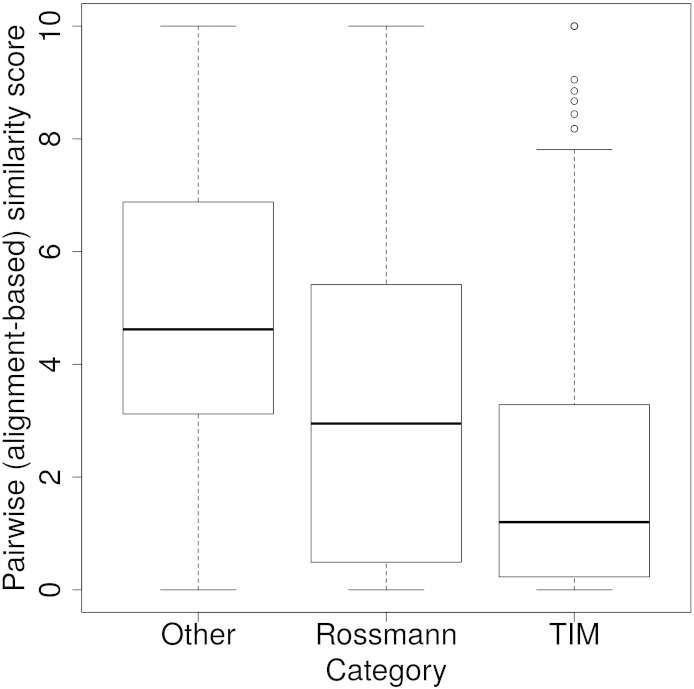
The distribution of pairwise catalytic residue similarity scores across alpha/beta superfamilies. The range of pairwise catalytic residue similarity scores for (1) superfamilies with a TIM barrel fold (CATH ID 3.20.20), (2) superfamilies with a Rossmann fold (CATH ID 3.40.50), and (3) all other superfamilies in the alpha/beta CATH class. The similarity scores have been generated through the structure-based, sequence-alignment-based protocol.

Nearly 72% of the 101 enzyme superfamilies have at least two functional families with different catalytic residues. A large proportion of functional family pairs (527 out of 785, 67%) have a CMSS of 5 or less (out of 10). Of these, 71.54% are from superfamilies with either a triosephosphate isomerase (TIM) or a Rossmann fold, which are significantly more diverse than other folds, with median CMSS values of 1.2 and 2.95, respectively (see Fig. S14), compared to 4.62 for all other superfamilies in the alpha/beta class (*P* value < 2.2 × 10^− 16^ using a Welch one-way ANOVA test). This is also accompanied by a greater variation in enzyme function [measured by calculating the average number of different EC numbers (at the third EC hierarchical level, i.e., 1.1.1) per superfamily in the Rossmann (12 EC 3 on average) or TIM (12 EC 3 on average) fold compared to remaining enzyme superfamilies (5 EC 3 on average)]. These observations are in agreement with recent analyses [26] suggesting that certain “innovable” folds, which include the TIM barrel or Rossmann fold, are more susceptible to functional divergence because their active sites comprise catalytic residues located on loops loosely connected to a well-structured, stable protein core.

**Fig. S15:**
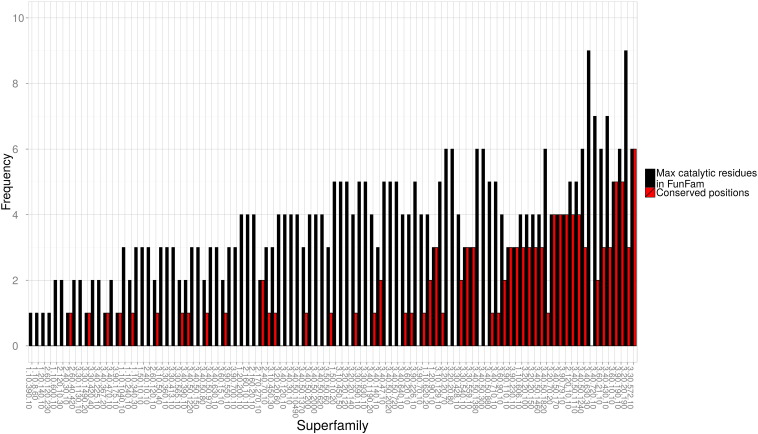
Exploring catalytic residue conservation across each superfamily. The maximum number of catalytic residues in a functional family is compared with the number of completely conserved catalytic residue positions across a particular superfamily.

Although nearly three-quarters of the superfamilies showed changes in their catalytic residues in some functional families, we found that 40 out of the 101 superfamilies examined (39.6%) have at least one completely conserved catalytic residue found in equivalent positions in the sequence in all their functional families (see Fig. S15). These residues may be essential for a common catalytic step conserved across the superfamily.

#### Convergent evolution of chemistry in superfamilies

**Fig. 6:**
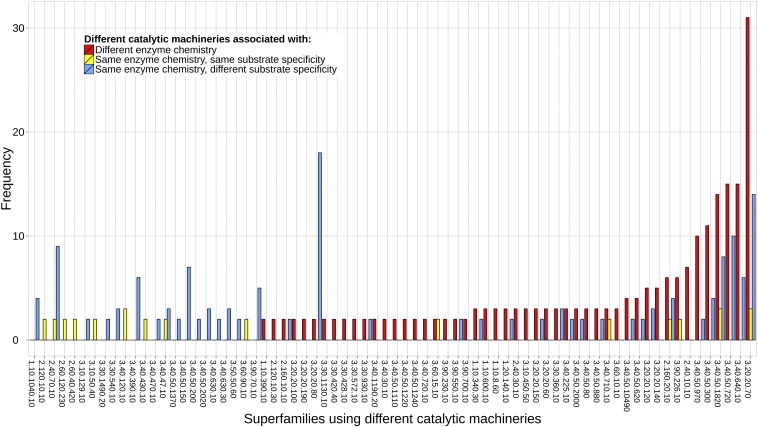
The number of cases in 72 superfamilies that use different catalytic machineries to (1) perform different enzyme chemistries, (2) perform the same enzyme chemistry with the same substrate specificity, and (3) perform the same enzyme chemistry with different substrate specificities. Catalytic machineries are defined as different when a pair of functional families has a catalytic residue similarity score of 5 or less. Differences in enzyme chemistry and substrate specificity are defined as changes at the third and fourth EC hierarchical levels, respectively.

A change in enzyme function can be dramatic, that is, a change in the chemistry performed and in the substrate. Whilst changes in catalytic residues usually result in changes in enzyme function (see Fig. 6), in most of these cases, the enzymes are performing the same chemistry (i.e., share the same EC number to the third level of the Enzyme Classification) on different substrates, and the residue changes we observe occur in residues that have less direct effects. For example, these residues may be influencing another residue or water molecule involved in catalysis or the binding of the substrate or co-factor involved in the reaction. Alternatively, they may be involved in stabilising a transition-state intermediate.

Perhaps most interesting are the functional families found in 16 of the superfamilies, which have different catalytic residues yet perform the same enzyme chemistry on the same substrate§. The exact evolutionary routes that led to these differences are often difficult to trace and would require a detailed analysis of the ancestral sequences and their functions. They may involve an intermediate with a different function or just a gradual change in the active site, whilst maintaining the original function. Recent work [27] using a detailed phylogenetic and experimental reconstruction of possible evolutionary pathways to trace the order of mutations and their impact on function in the mineralocorticoid receptor receptor family revealed the complexity of evolutionary paths, with tight restrictions on the order of residue changes.

### Examining the correlation between catalytic machinery and reaction mechanism

**Fig. S16:**
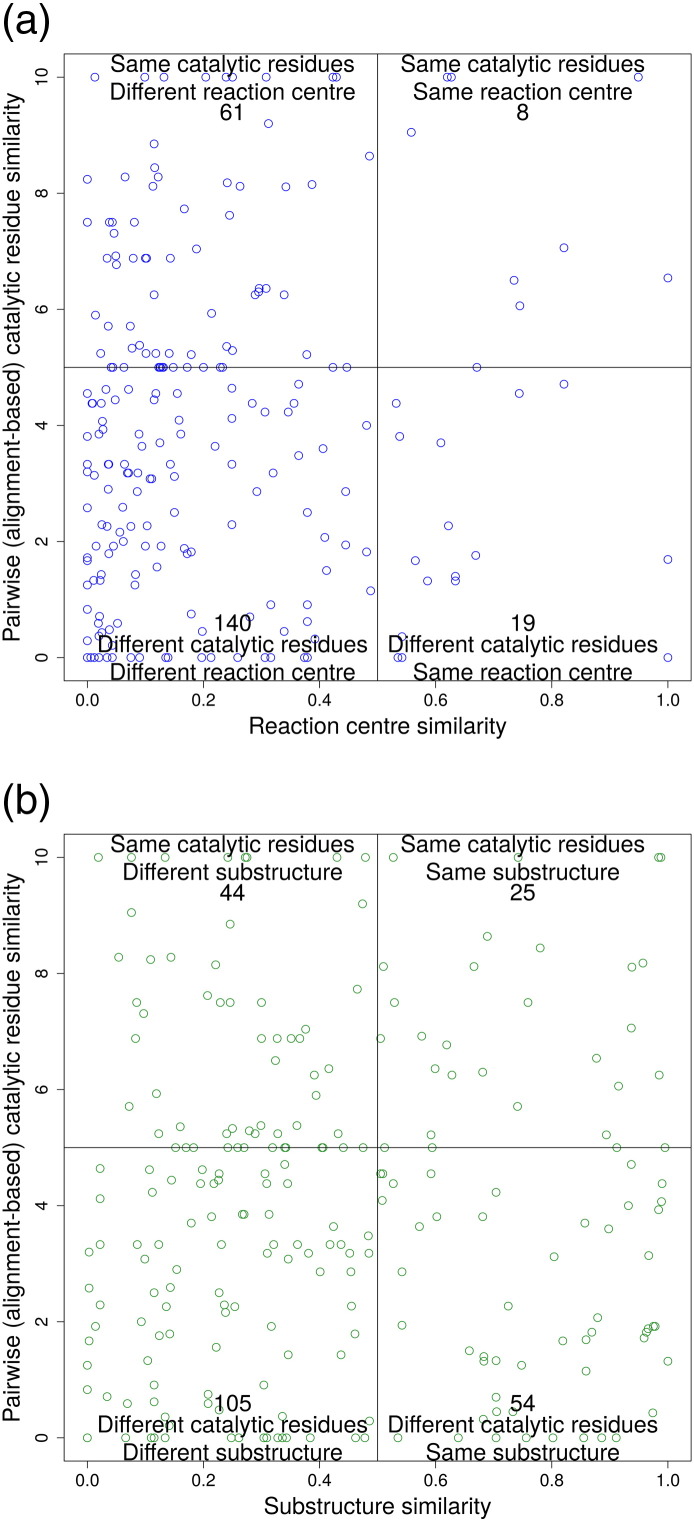
Examining whether there is a correlation between a change in catalytic machinery and reaction mechanism similarity. Catalytic residue similarity, calculated through the structure-based sequence alignment-based protocol, is plotted *versus* reaction centre similarity (a) and sub-structure similarity (b). The number in each quarter box represents the number of functional family pairs (i.e., points).

To examine the link between catalytic machinery and chemistry further, we also used EC-BLAST to determine whether a change in catalytic machinery was accompanied by a change in the reaction mechanism. EC-BLAST uses reaction mechanism information from IUBMB. We examined the bond change for 228 pairs of functional families for which there was sufficient information (correlations between similarity in catalytic machinery and similarity in reaction centre or small molecular sub-structure were also examined; see Fig. S16).

**Fig. 7:**
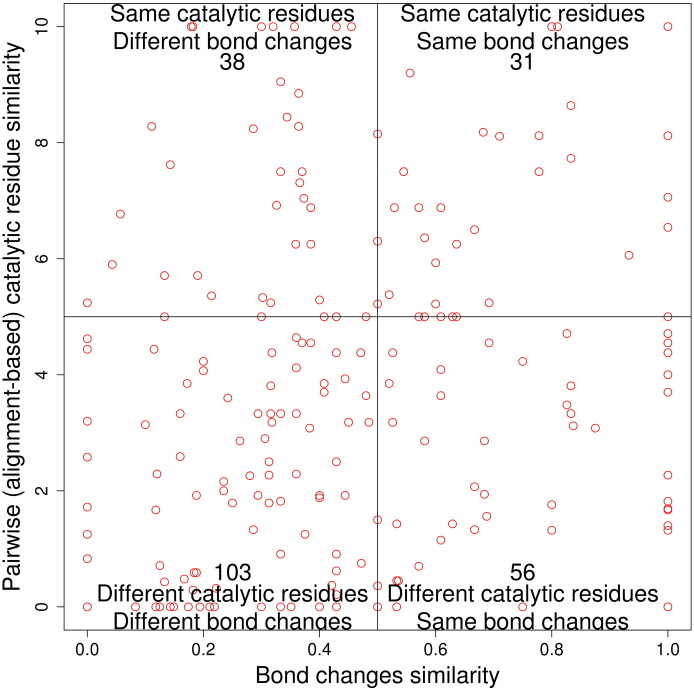
Examining whether there is a correlation between a change in catalytic machinery and reaction mechanism similarity. Catalytic residue similarity is plotted *versus* bond changes similarity. The number in each quarter box represents the number of functional family pairs (i.e., points).

As bond change represents the bonds formed and cleaved during a chemical reaction, we may expect some correlation between similarity in bond change and similarity in catalytic machinery. However, Fig. 7 shows no clear correlation, although 31 functional family pairs (13.60%) with similar catalytic machinery do exhibit similarity in bond change. Also, unsurprising is the greater density of points in the bottom-left quadrant (44% of functional family pairs), that is, where difference in catalytic residues is associated with difference in bond order change. The other quadrants in Fig. 7 are discussed in more detail below.

#### Same reaction mechanism, different catalytic machinery (bottom-right quadrant)

As discussed already, above, there are some superfamilies in which different catalytic machineries are supporting the same chemistry. In Fig. 7, there are 56 pairs of functional families (25%), which have different catalytic machineries but form/cleave the same bonds. We discuss some of the extreme outliers in this category below.

**Fig. 8:**
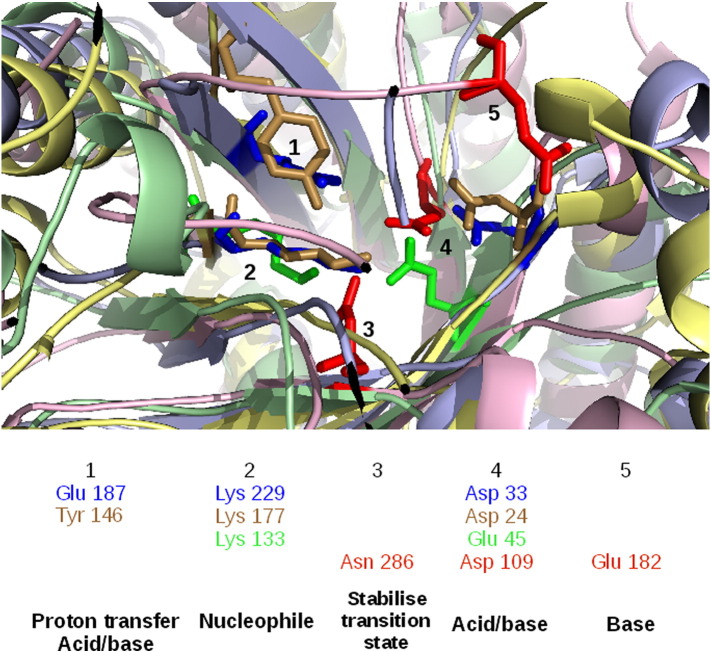
Comparing the structural positions and functional properties of catalytic residues in four domains with the same enzyme activities and different catalytic machineries. A superposition of CATH domains from the Aldolase Class I superfamily (ID 3.20.20.70) that catalyse aldehyde lyase activity (EC 4.1.2.-): 1aldA00 (light blue), 1ok4A00 (light yellow), 1fq0A00 (light green), and 1b57A00 (light pink). The catalytic residues from these four functional family representative domains cluster into five spatial sites and one can assign a common functional property to each cluster.

The “Aldolase Class I” CATH superfamily (CATH ID 3.20.20.70) has four functional families with the same reaction mechanisms (i.e., bond changes) but different catalytic machineries. Relatives in all four families have the same aldehyde lyase enzymatic activity and their catalytic residues co-locate in the same active site at the top of the beta barrel. Two of their three catalytic residues are found in loops, with the third residue in a beta strand, except for one family whose both the two catalytic residues are in beta strands. Although the catalytic residues occur in different positions in the sequence, the spatial sites of residues having similar chemical properties and catalytic roles are close in the structure (see Fig. 8). Although each functional family is binding a different substrate, the similarities in chemical properties and roles of the catalytic residues suggest a common mechanistic step that has been preserved amongst the relatives.

#### Different reaction mechanism, same catalytic machinery (top-left quadrant)

The top-left quadrant in Fig. 7 shows that a change in reaction mechanism is sometimes achieved without a large, or any, change in the catalytic machinery. There are 38 pairs (16.7%) of functional families that use similar, or sometimes identical, catalytic residues to catalyse different chemical reactions. Some of these cases arise because we examine catalytic machinery at the domain level but the function refers to the whole protein. However, other cases could be multi-functional proteins with the ability to be promiscuous.

**Fig. S17:**
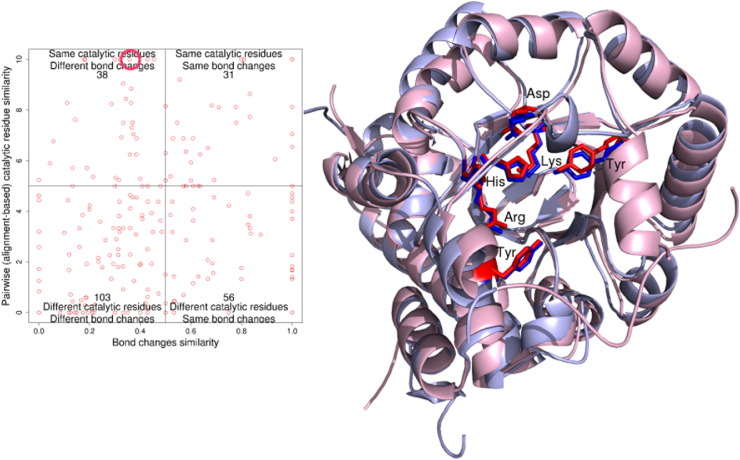
Two domains that catalyse different chemical reactions with the same catalytic machineries CATH domains 1fcbA02 (light blue) and 1goxA00 (light pink) from yeast l-lactase dehydrogenase (or FCB) (EC 1.1.2.3) and spinach GOX (EC 1.1.3.15), respectively, have been superimposed to show that they use the same catalytic machineries to catalyse different catalytic reactions.

**Fig. S18:**
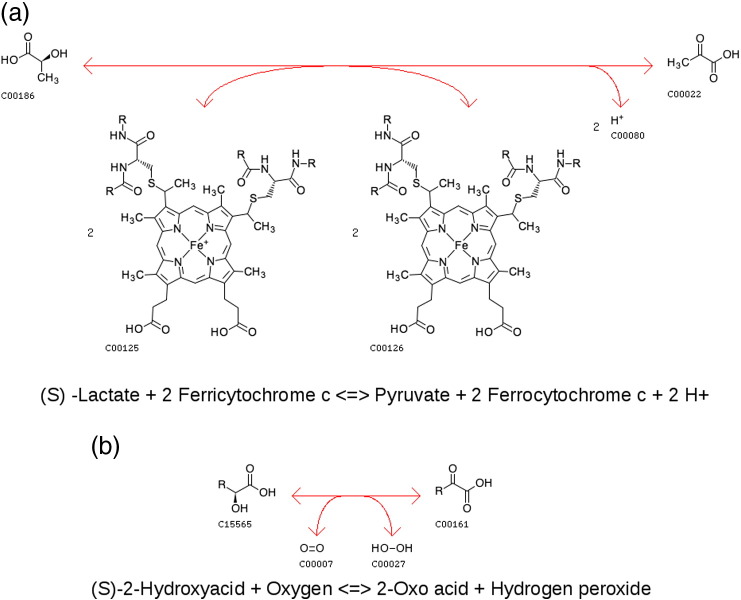
The different reaction mechanisms catalysed with the same catalytic machineries. The same catalytic residues at the same positions in structure are used by two protein domains to catalyse different reaction mechanisms. One enzyme, yeast l-lactase dehydrogenase (also known as FCB) is a dehydrogenase (EC 1.1.2.3) (a) whereas spinach GOX is an oxidase (EC 1.1.3.15) (b).

Yeast l-lactase dehydrogenase (also known as flavocytochrome *b*2 or FCB) and spinach glycolate oxidase (GOX) enzymes are two functional families from the same superfamily, where function changes are occurring within the same domain. FCB is a dehydrogenase (EC 1.1.2.3) whereas GOX is an oxidase (EC 1.1.3.15). Both are flavoprotein enzymes that catalyse the oxidation of different l-alpha-hydroxy acids. All relatives in the superfamily bind a flavin co-factor and have six conserved active-site residues [28], [29] (see Fig. S17 showing a superposition of the two domains). Interestingly, the first steps in both enzymatic reactions are similar in that the lactate substrate of FCB and the glycolate substrate of GOX are oxidised and the flavin mononucleotide (FMN) co-factor is reduced. Subsequently, the reactions diverge. For the FCB-catalysed reaction, the electrons from the FMN are used to reduce the iron atom in cytochrome *c* [30]. Whereas in the GOX-catalysed reaction, the electrons from the FMN are used to reduce oxygen to hydrogen peroxide [31] (see Fig. S18).

### Do catalytic residues generally locate to a particular part of the protein structure?

Although previous studies have examined the location of catalytic residues [4], we wanted to revisit this question with the much larger dataset available (i.e., 379 enzyme superfamilies compared to 31 [4]). We found that, of the three classes examined, superfamilies in the mainly beta class have the highest normalised proportion (NP) of catalytic residues in secondary structure (i.e., beta strands), whilst for superfamilies in the mainly alpha and alpha/beta classes, catalytic residues are mostly found in coil regions (see Table 1). Furthermore, the NP values for catalytic residues in coil regions in the alpha/beta class are significantly larger on average than in coil regions in the mainly alpha class (*P* value of 2.77E-11) and in the mainly beta class (*P* value of 1.98E-08).

A total of 91% (343) of the 379 enzyme superfamilies in this study are diverse in their substrate specificity (i.e., they contain at least two different EC 4 annotations). Since a large proportion of these functional diverse enzyme superfamilies (77%, 264) are alpha/beta, the high proportion of catalytic residues in loops in these folds lends some support to the hypothesis in Dellus-Gur *et al.* [26]. This hypothesis suggests that innovable (i.e., functionally diverse) families tend to be those in which catalytic residues mostly lie in coil regions detached from the main structural scaffold and thus more able to mutate without destabilising the protein. Indeed, the normalised values for catalytic residues in coil regions for superfamily members within the four innovable folds [26] are significantly larger on average than for all other superfamily members in our dataset (*P* value of 6.64E-06). However, we found no significant difference between the mean normalised values of catalytic residues in coil regions for superfamilies in the innovable folds and all other alpha/beta class enzyme superfamilies (*P* value of 0.08), suggesting that many members of these alpha/beta class superfamilies may also be considered innovable.

## Conclusion

As many others before us have observed, we find that the evolution of enzyme function is extremely complex. However, by being able to take a broad view across a large and wide range of enzyme containing domain superfamilies, we are able to demonstrate some shared aspects. By bringing together classical analysis of relationships between sequence and structure with new qualitative measures of similarities of function, we can observe how some superfamilies are able to change chemistry, sub-substrate specificity, or combinations of the two. By performing detailed structural analysis of active-site residues, we can determine whether these changes occur due to modifications in the catalytic machinery and identify relatives that appear to diverge and then converge again to perform similar functions, whilst others are able to adapt their function without major changes to the active site.

As with previous studies, we demonstrate that diverse relatives are more likely to be performing the same or similar chemistries on different substrates. However, dramatic changes in chemistry are observed. For nearly half the available superfamilies, at least one common catalytic residue was found in all relatives supporting the view that there is a tendency to conserve the chemistry. Some extreme cases of divergence in catalytic machinery may reflect the need to fine-tune the active-site residue repertoire to activate different substrates or stabilise different transition states. Alternately, cases where no similarity in catalytic machinery is observed may suggest very diverse evolutionary routes that converge on the same function or routes whereby divergence from a common catalytic machinery, perhaps resulting in a loss in efficiency in a particular relative, is followed by further mutations in different positions within the active site, giving rise to a different residue environment that has the ability to perform the same chemistry.

The observations that we report here are crucial to understanding the molecular basis of function evolution and furthering function prediction methods for the plethora of uncharacterized sequences and in the application of the development of novel synthetic enzymes in biotechnology.

## Materials and Methods

### Reconstructing enzyme functional changes in domain superfamilies

The protocol for generating multiple sequence alignments, phylogenetic trees, and associated changes in function is based on that used in the construction of FunTree [32], with the following adaptations:

#### Building alignments

In order to avoid the problems associated with aligning very diverse relatives, FunTree derives phylogenetic trees using a multiple sequence alignment that is guided by multiple structure alignment of structurally coherent relatives. Structurally coherent relatives are defined as those that superpose within 9 Å RMSD. CATH identifies structurally similar groups (SSGs) comprising relatives within a superfamily clustering with a threshold of 9 Å RMSD. Structural representatives from across the superfamily were selected from CATH functional families. Functional families are identified within each superfamily using a novel agglomerative clustering method that groups sequences sharing similar sequence patterns that relate to specificity determining positions in the family [33], [34]. CATH functional families have been shown to be much more structurally and functionally coherent than superfamilies and have performed well in protein function prediction [33], [35].

Compared to previous work that used representatives of sequence clusters at 35% sequence identity, building up the alignment from representatives of functionally coherent clusters of domain sequences permitted more sequences to be included in the alignment and reduced the number of SSGs representing a domain superfamily.

#### Tree generation

Phylogenetic trees for 379 structurally defined enzyme superfamilies defined by the CATH classification were generated using the CSA [12] and MACiE [11] databases to help define enzyme containing CATH domain superfamilies. Using the improved protocol described above for producing structurally informed multiple sequence alignments, we generated phylogenetic trees for each SSG in a superfamily. In some superfamilies, since the structurally informed multiple sequence alignments contained many thousands of sequences, a filtering algorithm was implemented to reduce the number of sequences to make the alignments amenable to phylogenetic analysis. Though sequences were removed, the alignment was not changed, with representatives displayed in the trees chosen to maximise functional and taxonomic diversity, structural coverage, and multi-domain architecture and to reduce functional repetition and closely related homologues. The trees were built using TreeBeST (Tree Building guided by Species Tree) [36], which employs a maximum-likelihood-based reconstruction method combined with a species tree based on the National Center for Biotechnology Information taxonomic definitions.

#### Ancestral character estimation

The enzyme functional annotations, combined with the phylogenetic tree, were used to infer the ancestral function at each node in the tree using the discrete ancestral character estimation algorithm with an equal rates model as implemented in the APE (Analyses of Phylogenetics and Evolution) [37] package in the R statistical suite. At each node in the tree, maximum-likelihood estimation is made of the most probable function. It should be noted that the ancestral function is assumed to be one of the modern functions observed at the leaves of the tree. This permits the functional changes from parent node to a child node to be traced through the tree and to catalogue the changes in function based on the EC number. Comparisons between parent and child reactions can be made using the EC-BLAST algorithm, using normalised bond order, reaction centre, and small molecule sub-structure similarity scores.

#### Measuring functional similarity

The protein domains at the leaves in each tree were annotated with EC numbers [38] obtained via the UniProtKB resource. Using the IUBMB reactions describing each of the EC numbers, we compared each reaction to each other within an SSG using the EC-BLAST [23] algorithm. Briefly, this uses atom–atom mapping to derive knowledge of bond changes and reaction patterns for all known biochemical reactions, using a variation of the Dugundji–Ugi matrix model. Comparisons were made using three types of normalised similarity scores. The first, bond order, compares the changes in the number and type of bonds that are being broken and formed. Secondly, the reaction centre metric compares the local chemical environment around the centre of the reaction; that is, the atoms covalently linked to the atoms forming the bond that is broken/formed in a reaction (Fig. S19). Finally, the substrates and products of the reactions are compared using a common sub-graph detection algorithm implemented in SMSD (Small Molecule Subgraph Detector) that identifies similar fragments from all the metabolites in a reaction [39].

**Fig. S20:**
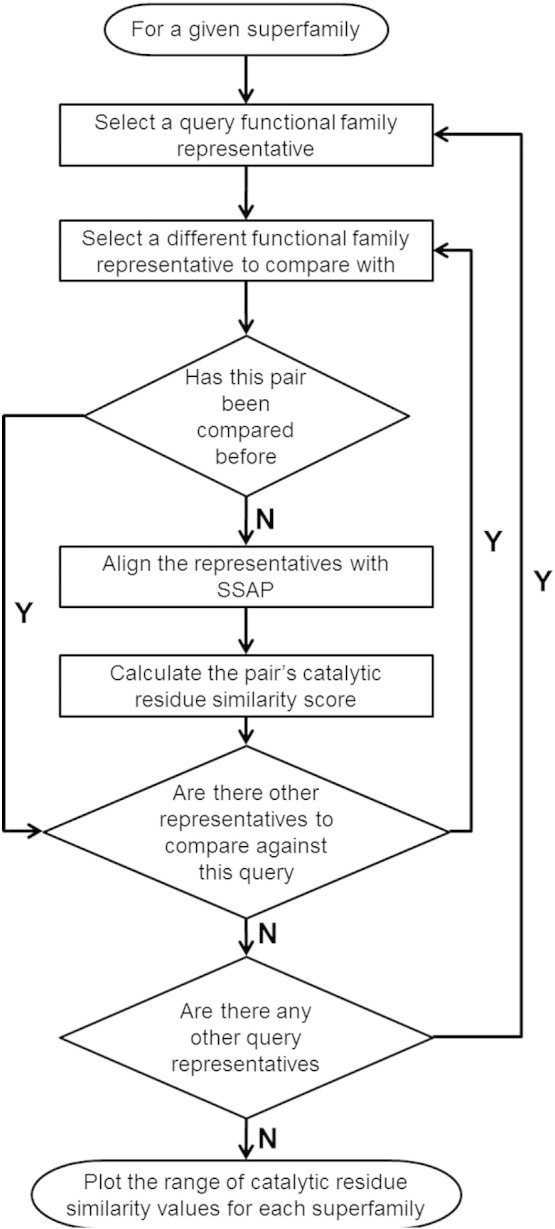
A summary flowchart of the protocol employed in cataloguing changes in function.

A summary|| of the protocol used is provided in Fig. S20.

### Examining changes in catalytic machinery across enzyme domain superfamilies

The CSA stores information on catalytic site residues derived from the literature and also from homology searches, and it defines them as a residue (1) with direct involvement in the catalytic mechanism, (2) affecting another residue or water molecule directly involved in the catalytic mechanism, (3) that stabilises a transition-state intermediate, and (4) that exerts an effect on a substrate/co-factor aiding catalysis. Residues involved in ligand binding are excluded unless they are involved in one of the above four functions [40].

Structural domains in functional families were annotated with CSA functional residues that had literature-based evidence. A functional family representative was chosen, by selecting the structural domain annotated with the most CSA functional residues.

A subset of the 379 CATH version 3.5 enzyme superfamilies was created containing 101 superfamilies with CSA data for two or more functional families in order to make comparisons between at least two sets of experimentally validated catalytic sites in a superfamily.

**Fig. 9:**
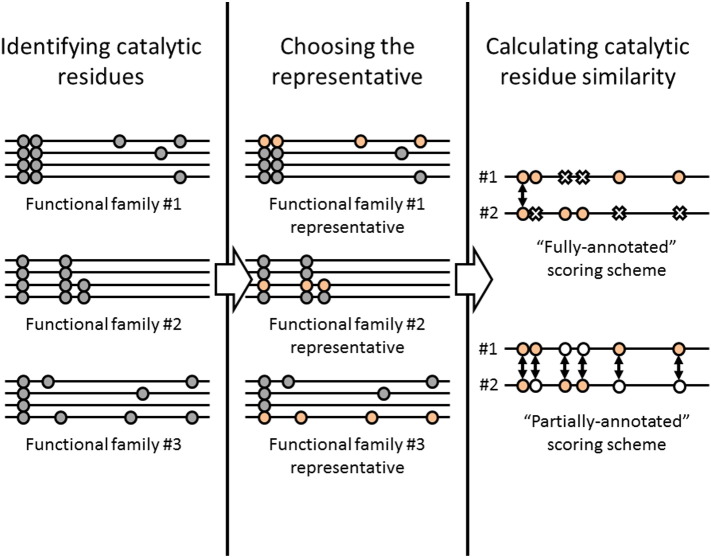
Calculating catalytic residue similarity between functional families catalytic residues from the CSA are identified within each structural domain in a functional family where available (1). The functional family representative is then chosen as the structural domain with the highest number of catalytic residues identified (2). Pairs of functional family representatives within a superfamily are aligned using SSAP and their catalytic residue similarity scored. The “fully-annotated” scoring scheme only takes into account pairs of CSA-annotated residues. The “partially-annotated” scoring scheme scores pairs of residues where at least one residue has a CSA annotation.

To compare the catalytic residues between two relatives, we pairwise aligned all functional family representative domains within each superfamily in the dataset with SSAP (Sequential Structure Alignment Program) [41]. For relatives whose structures aligned well (i.e., with an RMSD of ≤ 5 Å), equivalent positions were compared in the alignment where at least one of the residues was a catalytic residue (see Fig. 9 and Fig. S21). A quantitative measure of similarity between the residues in equivalent positions was calculated based on the physicochemical similarity scoring matrix [42] that compares amino acids based upon their amino acid polarity, size, shape, and charge. This measure was normalised to give a scoring range of 0 (minimum difference) to 10 (maximum difference). Two scoring schemes were used: (1) the “fully-annotated” approach where physicochemical similarity of the aligned residues was scored if both were annotated as catalytic, and (2) the “partially-annotated” approach where at least one residue should be annotated as being catalytic for an equivalent position to be scored. The latter accounts for missing annotations or mis-annotations. In both schemes, a catalytic residue aligned to a gapped position was penalised with the lowest score of 0. Scores were accumulated across the catalytic residue positions in an alignment and divided by the number of positions scored.

### Examining the structural preference of catalytic residues

The structural location of catalytic residues was examined using all the 379 superfamilies in the original dataset. The secondary structure of each catalytic residue was assigned using the DSSP programme and the BioPerl DSSP module [43] into four categories: helix (H, G, and I), beta structure (B and E), turn (T and S), and no assignment (i.e., loop regions). The turn and no assignment categories were merged and will be referred to as coil regions.

The proportion of catalytic residues in each domain examined was calculated in each of these three assigned categories and normalised by the proportion of all residues in each of the categories [see Eq. (1) below].

$$NP=\left(\frac{C_{\mathrm{category}}}{C_{\mathrm{total}}}\right)\times \;\left(1-\frac{R_{\mathrm{category}}}{R_{\mathrm{total}}}\right)$$

Eq. (1) is the NP of catalytic residues in each secondary structure category examined.

For a given domain, *C*_category_ represents the number of catalytic residues in a particular secondary structure category, *C*_total_ represents the total number of catalytic residues, *R*_category_ represents the total number of residues in a particular secondary structure category, and *R*_total_ represents the total number of residues.

The Wilcoxon rank-sum test in R was used to assess whether there was a statistically significant difference between the distributions of normalised values for catalytic residues in coil regions for superfamily members in each of the three major CATH classes (alpha, beta, or alpha/beta).

To explore whether catalytic residues preferentially occur in the coil regions of innovative or functionally diverse superfamily members, we used the Wilcoxon rank-sum test to compare the distributions of catalytic residue proportions between “innovable” and “all other” superfamilies. The innovable superfamilies were defined as those belonging to the four folds described by Dellus-Gur *et al*. [26]: TIM barrel (CATH ID 3.20.20), Rossmann (CATH ID 3.40.50), Aminopeptidase (CATH ID 3.40.630), and Alpha/Beta Plaits (CATH ID 3.30.70).

The following are the supplementary data related to this article.

Fig. S1 A summary of changes involving oxidoreductases. All EC numbers in FunTree represented as nodes in a network ordered by EC class. Each change in function associated with a change to/from an oxidoreductase (as highlighted in the EC exchange matrix top right) is shown as an edge in the network (top left). The edges in the networks in the bottom row show the bond change, reaction centre, and sub-structure similarities, respectively, coloured using a grey scale where white is zero similarity and black is exactly the same.

Fig. S2 A summary of changes involving transferases. All EC numbers in FunTree represented as nodes in a network ordered by EC class. Each change in function associated with a change to/from an transferase (as highlighted in the EC exchange matrix top right) is shown as an edge in the network (top left). The edges in the networks in the bottom row show the bond change, reaction centre, and sub-structure similarities, respectively, coloured using a grey scale where white is zero similarity and black is exactly the same.

Fig. S3 A summary of changes involving hydrolases. All EC numbers in FunTree represented as nodes in a network ordered by EC class. Each change in function associated with a change to/from an hydrolase (as highlighted in the EC exchange matrix top right) is shown as an edge in the network (top left). The edges in the networks in the bottom row show the bond change, reaction centre, and sub-structure similarities, respectively, coloured using a grey scale where white is zero similarity and black is exactly the same.

Fig. S4 A summary of changes involving lyases. All EC numbers in FunTree represented as nodes in a network ordered by EC class. Each change in function associated with a change to/from a lyase (as highlighted in the EC exchange matrix top right) is shown as an edge in the network (top left). The edges in the networks in the bottom row show the bond change, reaction centre, and sub-structure similarities, respectively, coloured using a grey scale where white is zero similarity and black is exactly the same.

Fig. S5 A summary of changes involving isomerases. All EC numbers in FunTree represented as nodes in a network ordered by EC class. Each change in function associated with a change to/from an isomerase (as highlighted in the EC exchange matrix top right) is shown as an edge in the network (top left). The edges in the networks in the bottom row show the bond change, reaction centre, and sub-structure similarities, respectively, coloured using a grey scale where white is zero similarity and black is exactly the same.

Fig. S6 A summary of changes involving ligases. All EC numbers in FunTree represented as nodes in a network ordered by EC class. Each change in function associated with a change to/from an ligase (as highlighted in the EC exchange matrix top right) is shown as an edge in the network (top left). The edges in the networks in the bottom row show the bond change, reaction centre, and sub-structure similarities, respectively, coloured using a grey scale where white is zero similarity and black is exactly the same.

Fig. S7 Comparing bond order similarities by EC class. All EC numbers in FunTree represented as nodes in a network ordered by EC class. Each change in function associated with a change to/from each EC class is shown in each of the networks as an edge. The edge colour shows the reaction centre similarity score using a grey scale where white is zero similarity and black is exactly the same.

Fig. S8 Comparing reaction centre similarities by EC class. All EC numbers in FunTree represented as nodes in a network ordered by EC class. Each change in function associated with a change to/from each EC class is shown in each of the networks as an edge. The edge colour shows the reaction centre similarity score using a grey scale where white is zero similarity and black is exactly the same.

Fig. S9 Comparing sub-structure similarities by EC class. All EC numbers in FunTree represented as nodes in a network ordered by EC class. Each change in function associated with a change to/from each EC class is shown in each of the networks as an edge. The edge colour shows the sub-structure similarity score using a grey scale where white is zero similarity and black is exactly the same.

Fig. S10 Summary of Vanillyl-Alcohol Oxidase superfamily (CATH ID 1.10.45.10). (a) Overall reaction diagrams with sub-structures identified by SMSD coloured for the two reactions found in the superfamily: vanillyl-alcohol oxidase (EC 1.1.3.38) and 4-methylphenol dehydrogenase (EC 1.17.99.2). (b) A summary table of bond changes for each reaction.

Fig. S11 Summary of Amidase Signature enzyme superfamily (CATH ID 3.90.1300.10) reactions. (a) Overall reaction diagrams with sub-structures identified by SMSD coloured for two of the reactions found in the superfamily: amidase (EC 3.5.1.4) and 6-aminohexanoate-cyclic-dimer hydrolase (EC 3.5.2.12). (b) A summary table of bond changes for each reaction. (c) A summary table of conservation of catalytic residues in the sequences of the other enzymes in the superfamily, with a tick indication conservation and a cross indication that it is not conserved.

Fig. S12 Conservation of bond types between changes in function. The top 10 cumulative counts of bond types that remain the same between changes in function across all the changes observed in FunTree. The inset shows the distribution of the top 20 bond changes across all known reactions.

Fig. S19 Reaction centre. A first-order reaction centre is defined as the atoms involved in the bond change and those atoms directly connected to them. The reaction centre is highlighted in grey and the atoms that make up the reaction centre are highlighted in red.

Fig. S21 Flowchart of catalytic residue similarity analysis. The steps taken in calculating catalytic residue similarity values for all pairs of functional families in each superfamily analysed.

Table S22 Summary of specificity compared to overall reaction chemistry for each superfamily. An overall description of specificity compared to overall reaction chemistry is provided with to the CATH superfamily pages for further exploration of functional changes.

## Figures and Tables

**Table 1:** The mean (normalised) value of catalytic residues found in different types of secondary structure and in coil regions for enzyme superfamilies in the three main CATH classes

	Mean (normalised) value of catalytic residues in		
	alpha Helix	beta Structure	Coil
Superfamilies in alpha class	0.22	0.05	0.23
Superfamilies in beta class	0.09	0.26	0.22
Superfamilies in alpha/beta class	0.14	0.18	0.31

## Footnotes

§ Alignments of active-site residues across each superfamily analysed can be viewed on http://www.cathdb.info/nataliedawson/csashift/list_all_superfamilies.

|| All the data used in this analysis can be found at http://www.ebi.ac.uk/thornton-srv/databases/FunTreeJMB.
