# Supplementary figures and images for: Large-Scale Analysis Exploring Evolution of Catalytic Machineries and Mechanisms in Enzyme Superfamilies
Source: J Mol Biol. 2016 Jan 29;428(2Part A):253–67. doi: 10.1016/j.jmb.2015.11.010 (PMC4751976; doi:10.1016/j.jmb.2015.11.010)

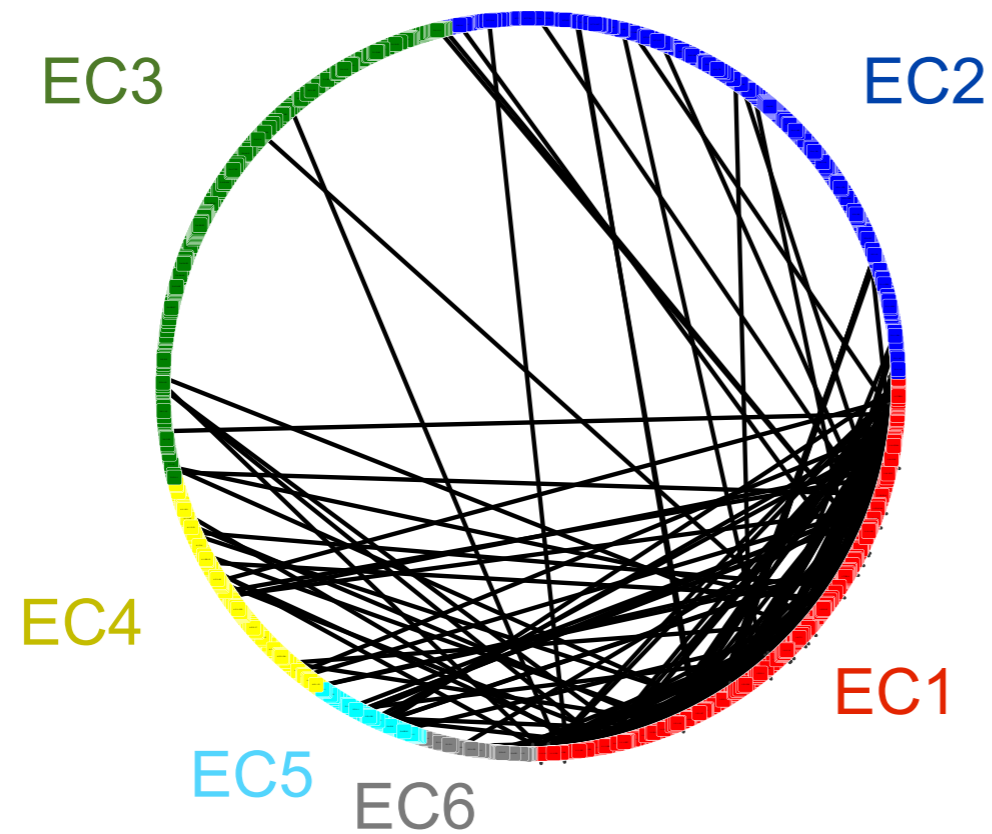

| 1    | 2   | 3   | 4   | 5  | 6   | E.C. Class |
|------|-----|-----|-----|----|-----|------------|
| 1014 | 37  | 23  | 27  | 16 | 15  | 1          |
| 44   | 934 | 91  | 78  | 33 | 16  | 2          |
| 21   | 79  | 928 | 36  | 19 | 5   | 3          |
| 16   | 38  | 17  | 191 | 32 | 5   | 4          |
| 10   | 19  | 10  | 33  | 52 | 0   | 5          |
| 13   | 14  | 7   | 7   | 1  | 213 | 6          |

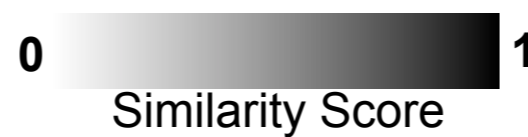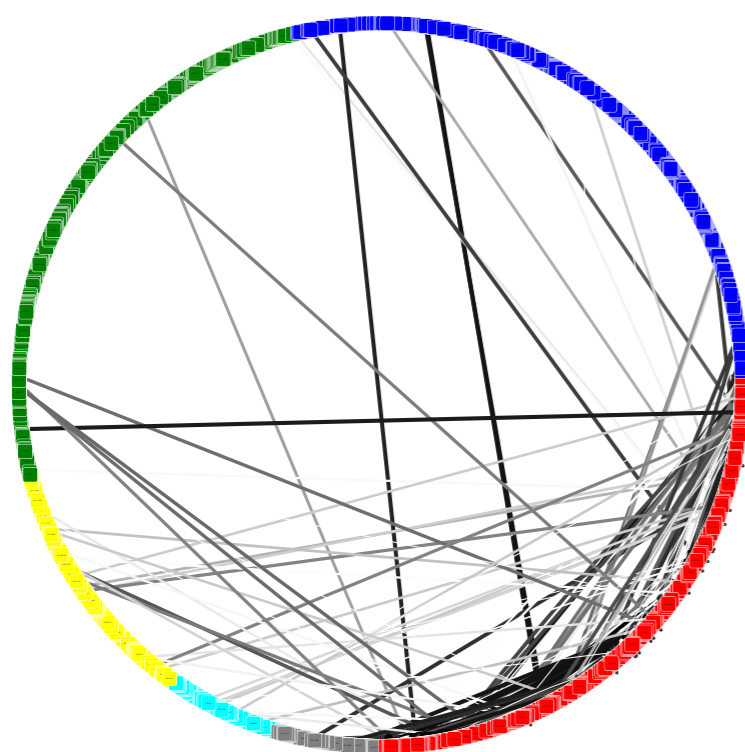

Bond Similarity

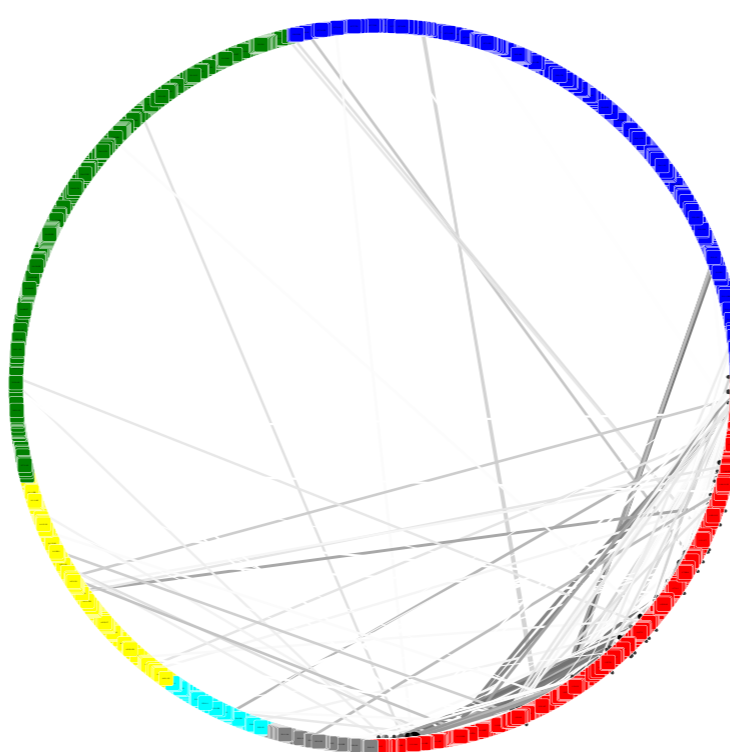

Reaction Centre Similarity

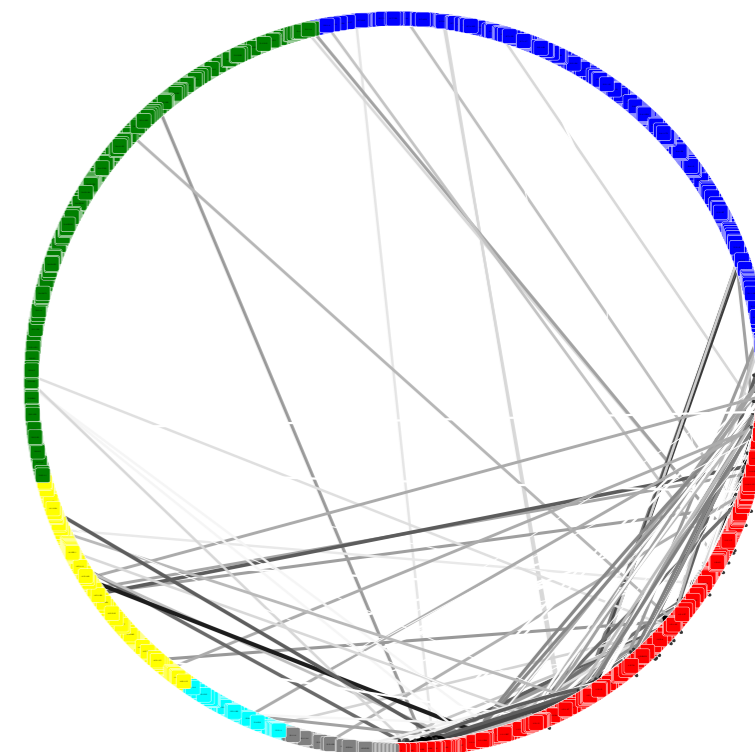

Sub-Structure Similarity

Supplement: Fig. S1 — A summary of changes involving oxidoreductases. All EC numbers in FunTree represented as nodes in a network ordered by EC class. Each change in function associated with a change to/from an oxidoreductase (as highlighted in the EC exchange matrix top right) is shown as an edge in the network (top left). The edges in the networks in the bottom row show the bond change, reaction centre, and sub-structure similarities, respectively, coloured using a grey scale where white is zero similarity and black is exactly the same. [file mmc1.pdf]

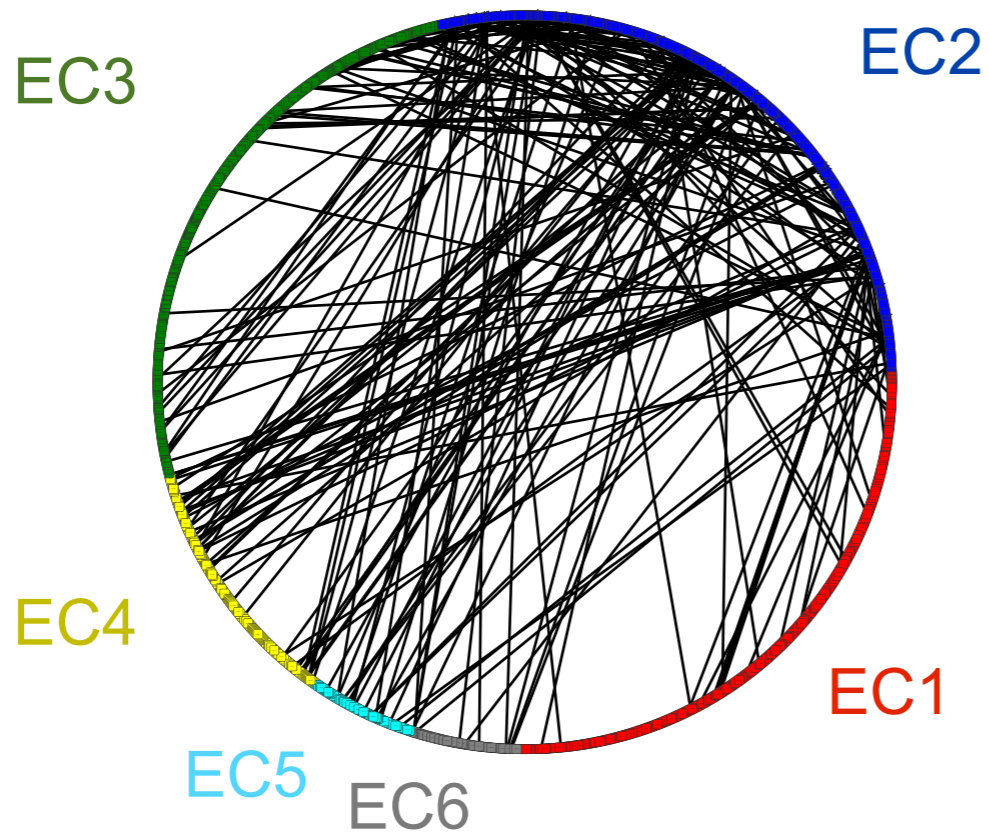

|      |     |     |     |    |     |            |
|------|-----|-----|-----|----|-----|------------|
| 1014 | 37  | 23  | 27  | 16 | 15  | 1          |
| 44   | 934 | 91  | 78  | 33 | 16  | 2          |
| 21   | 79  | 928 | 36  | 19 | 5   | 3          |
| 16   | 38  | 17  | 191 | 32 | 5   | 4          |
| 10   | 19  | 10  | 33  | 52 | 0   | 5          |
| 13   | 14  | 7   | 7   | 1  | 213 | 6          |
| 1    | 2   | 3   | 4   | 5  | 6   | E.C. Class |

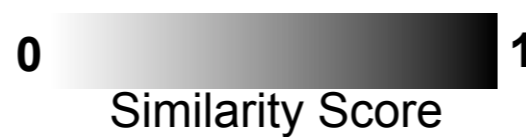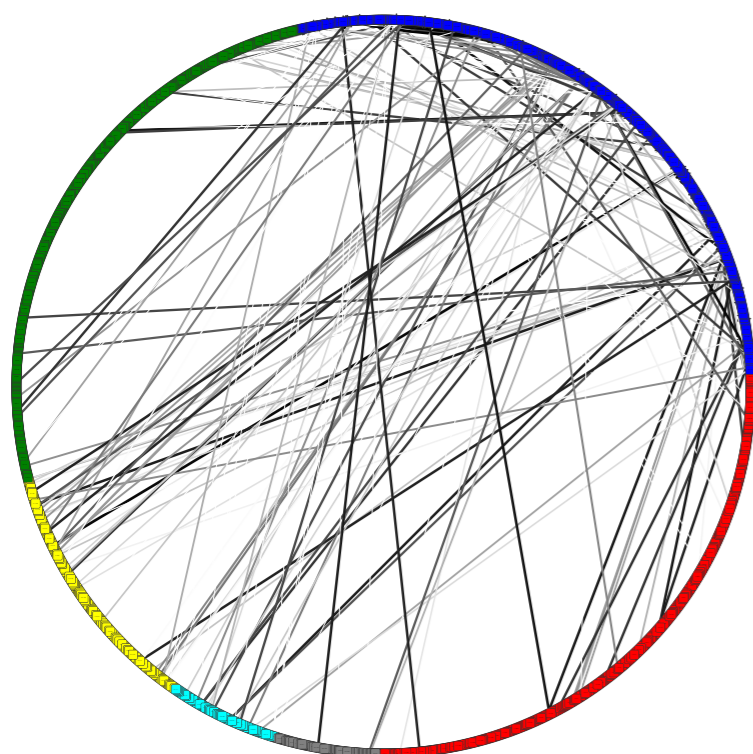

Bond Similarity

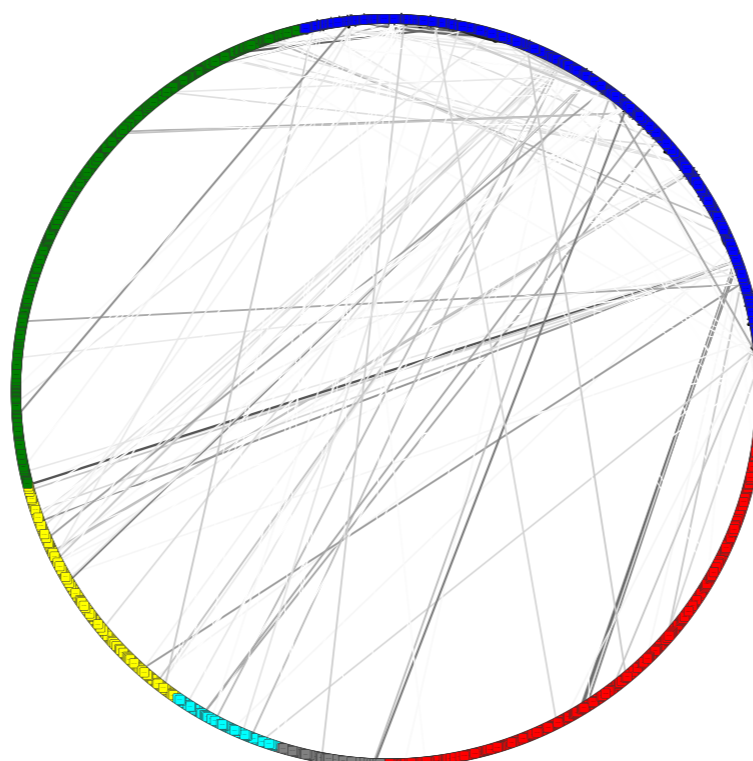

Reaction Centre Similarity

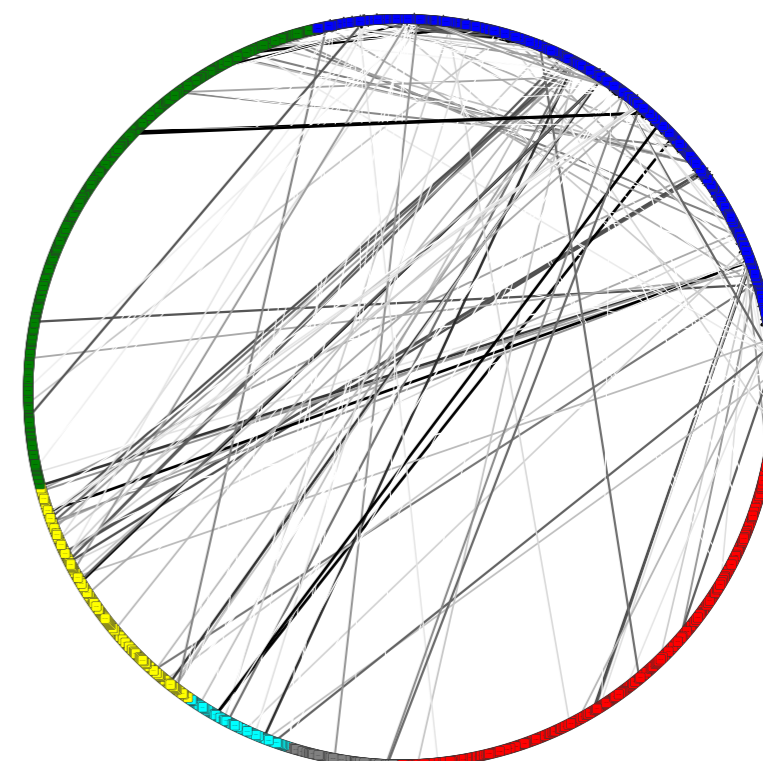

Sub-Structure Similarity

Supplement: Fig. S2 — A summary of changes involving transferases. All EC numbers in FunTree represented as nodes in a network ordered by EC class. Each change in function associated with a change to/from an transferase (as highlighted in the EC exchange matrix top right) is shown as an edge in the network (top left). The edges in the networks in the bottom row show the bond change, reaction centre, and sub-structure similarities, respectively, coloured using a grey scale where white is zero similarity and black is exactly the same. [file mmc2.pdf]

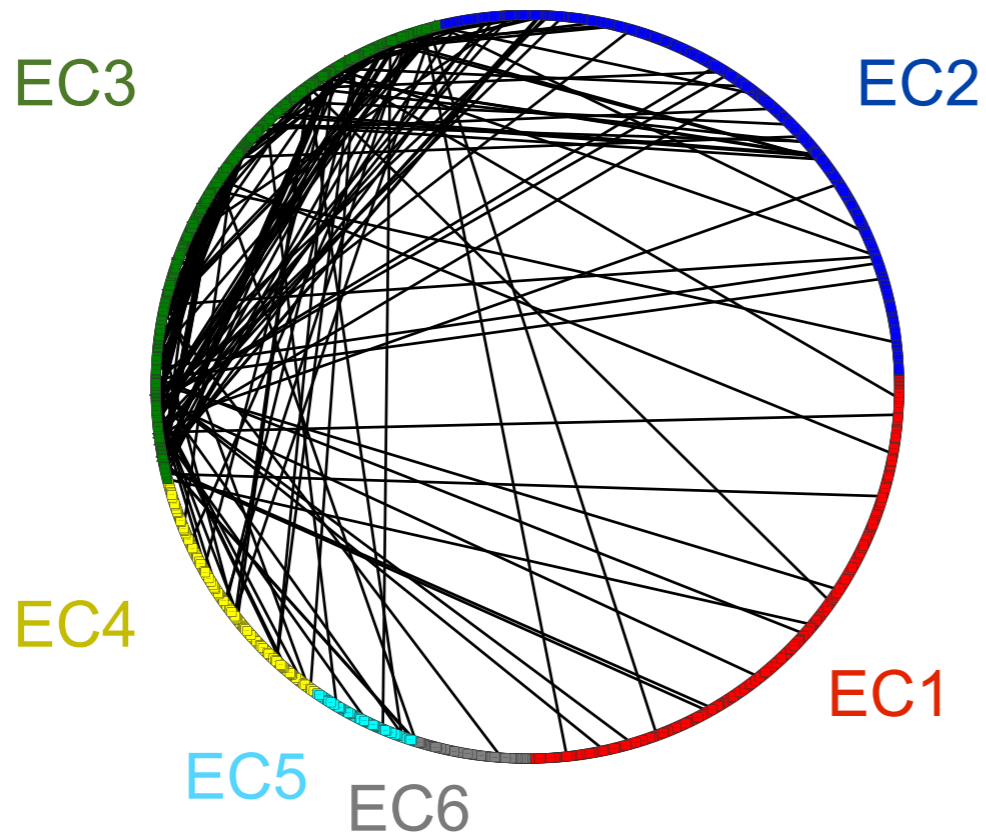

|      |     |     |     |    |     |            |
|------|-----|-----|-----|----|-----|------------|
| 1014 | 37  | 23  | 27  | 16 | 15  | 1          |
| 44   | 934 | 91  | 78  | 33 | 16  | 2          |
| 21   | 79  | 928 | 36  | 19 | 5   | 3          |
| 16   | 38  | 17  | 191 | 32 | 5   | 4          |
| 10   | 19  | 10  | 33  | 52 | 0   | 5          |
| 13   | 14  | 7   | 7   | 1  | 213 | 6          |
| 1    | 2   | 3   | 4   | 5  | 6   | E.C. Class |

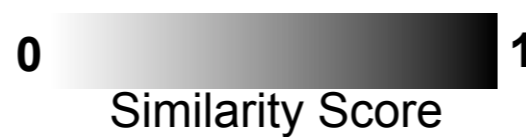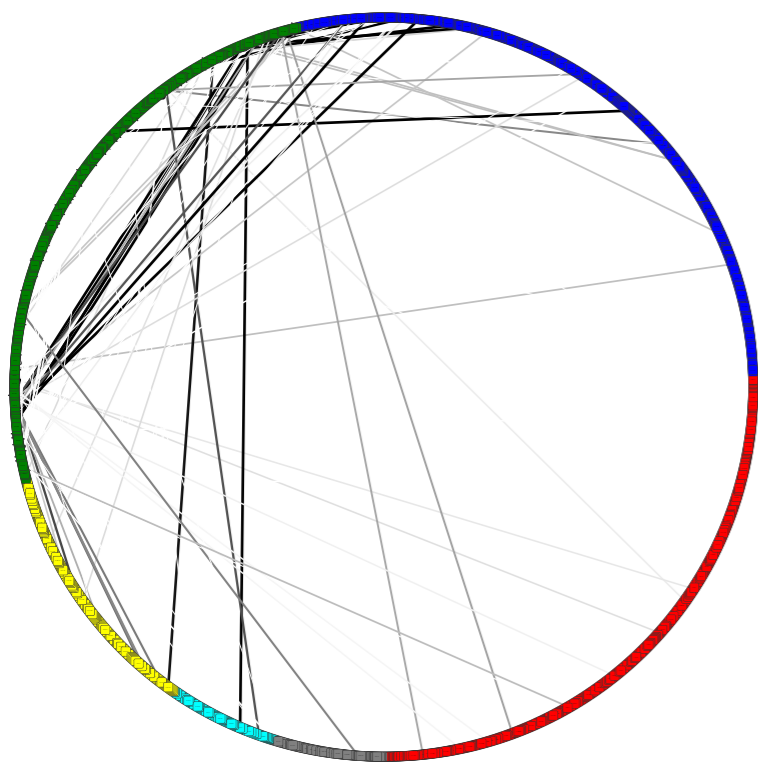

Bond Similarity

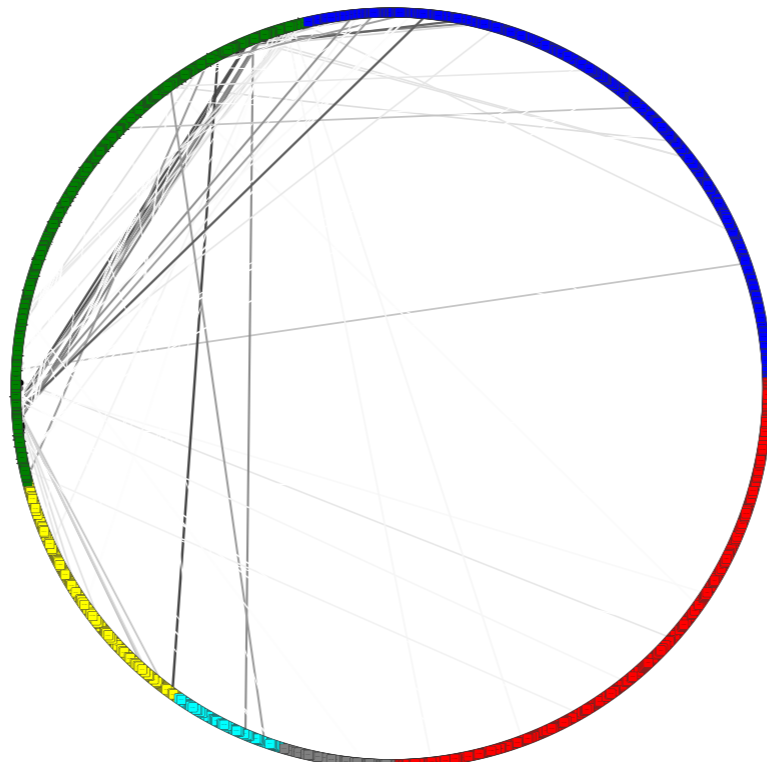

Reaction Centre Similarity

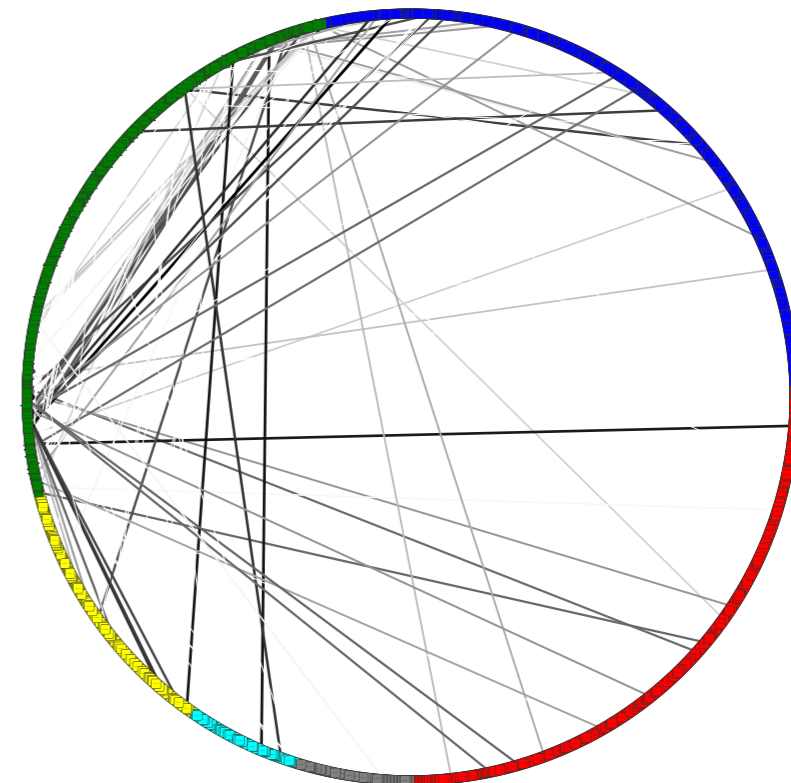

Sub-Structure Similarity

Supplement: Fig. S3 — A summary of changes involving hydrolases. All EC numbers in FunTree represented as nodes in a network ordered by EC class. Each change in function associated with a change to/from an hydrolase (as highlighted in the EC exchange matrix top right) is shown as an edge in the network (top left). The edges in the networks in the bottom row show the bond change, reaction centre, and sub-structure similarities, respectively, coloured using a grey scale where white is zero similarity and black is exactly the same. [file mmc3.pdf]

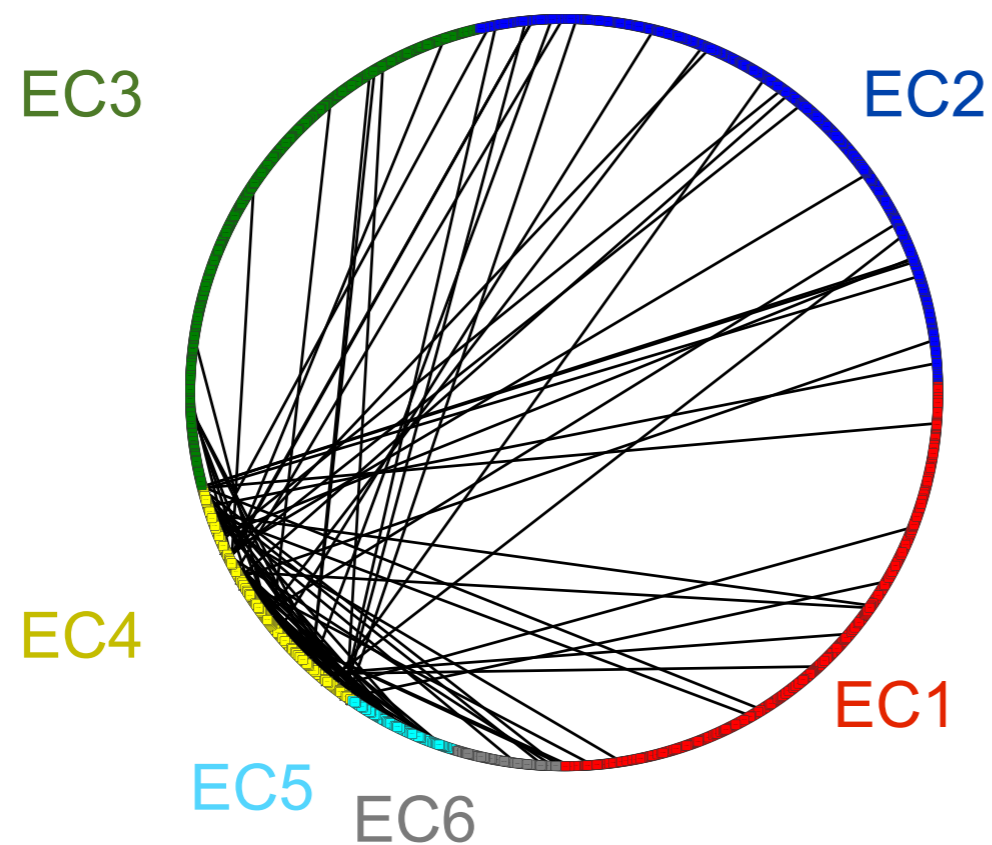

|      |     |     |     |    |     |            |
|------|-----|-----|-----|----|-----|------------|
| 1014 | 37  | 23  | 27  | 16 | 15  | 1          |
| 44   | 934 | 91  | 78  | 33 | 16  | 2          |
| 21   | 79  | 928 | 36  | 19 | 5   | 3          |
| 16   | 38  | 17  | 191 | 32 | 5   | 4          |
| 10   | 19  | 10  | 33  | 52 | 0   | 5          |
| 13   | 14  | 7   | 7   | 1  | 213 | 6          |
| 1    | 2   | 3   | 4   | 5  | 6   | E.C. class |

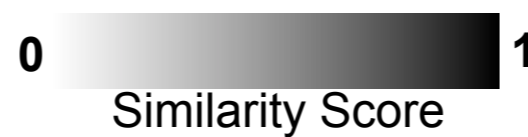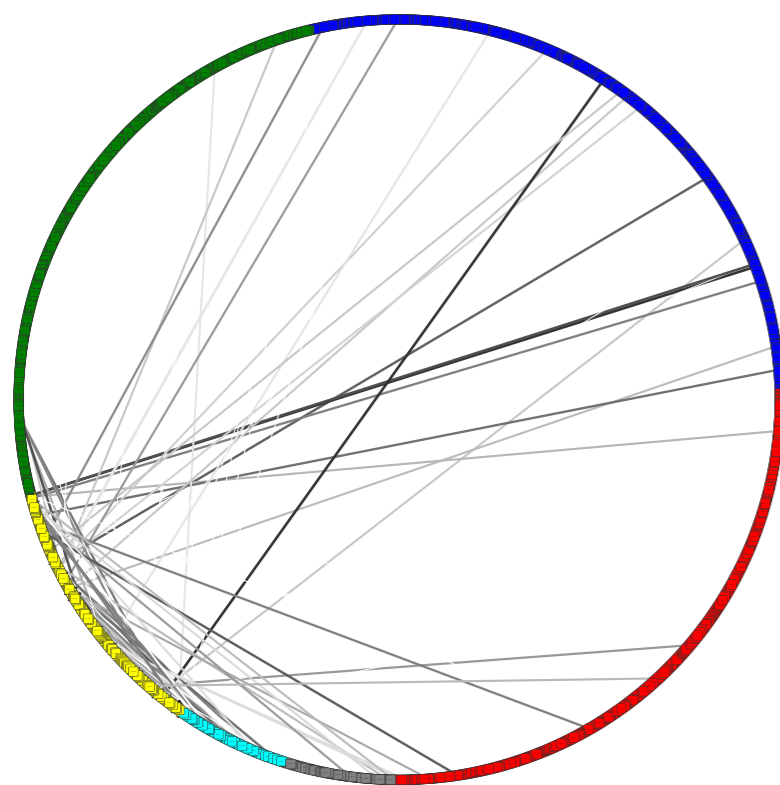

Bond Similarity

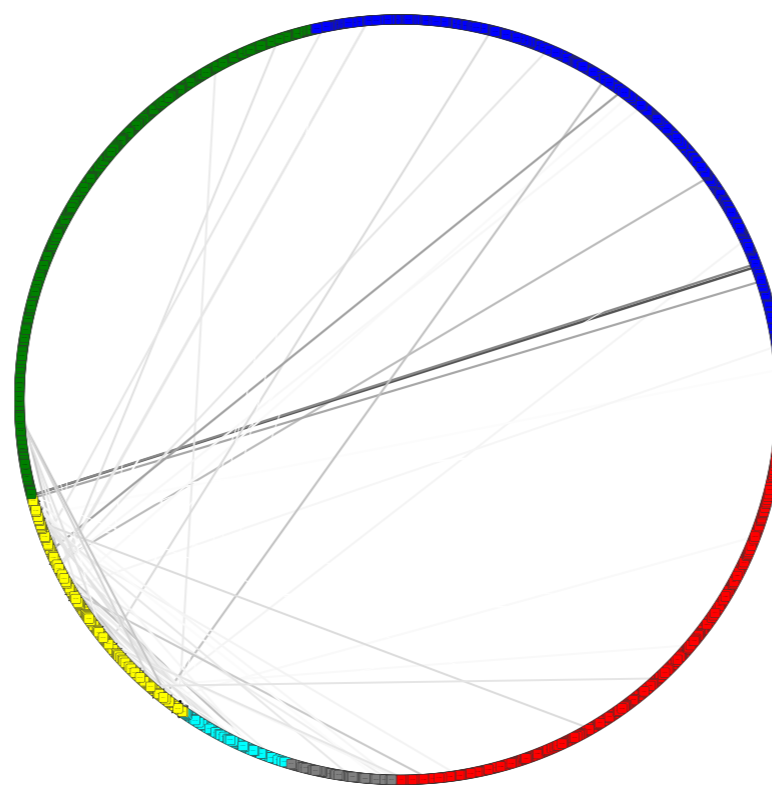

Reaction Centre Similarity

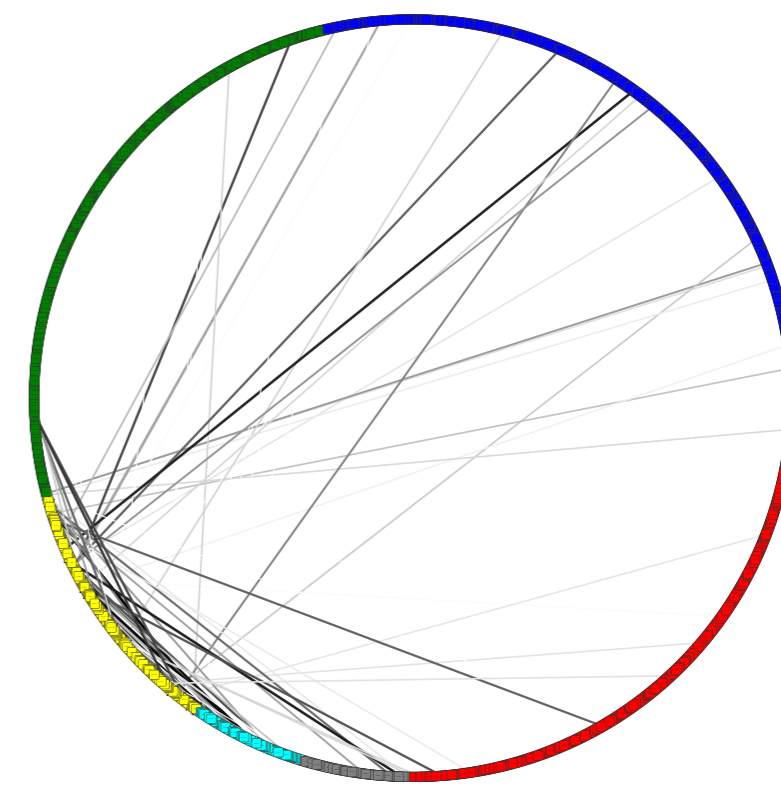

Sub-Structure Similarity

Supplement: Fig. S4 — A summary of changes involving lyases. All EC numbers in FunTree represented as nodes in a network ordered by EC class. Each change in function associated with a change to/from a lyase (as highlighted in the EC exchange matrix top right) is shown as an edge in the network (top left). The edges in the networks in the bottom row show the bond change, reaction centre, and sub-structure similarities, respectively, coloured using a grey scale where white is zero similarity and black is exactly the same. [file mmc4.pdf]

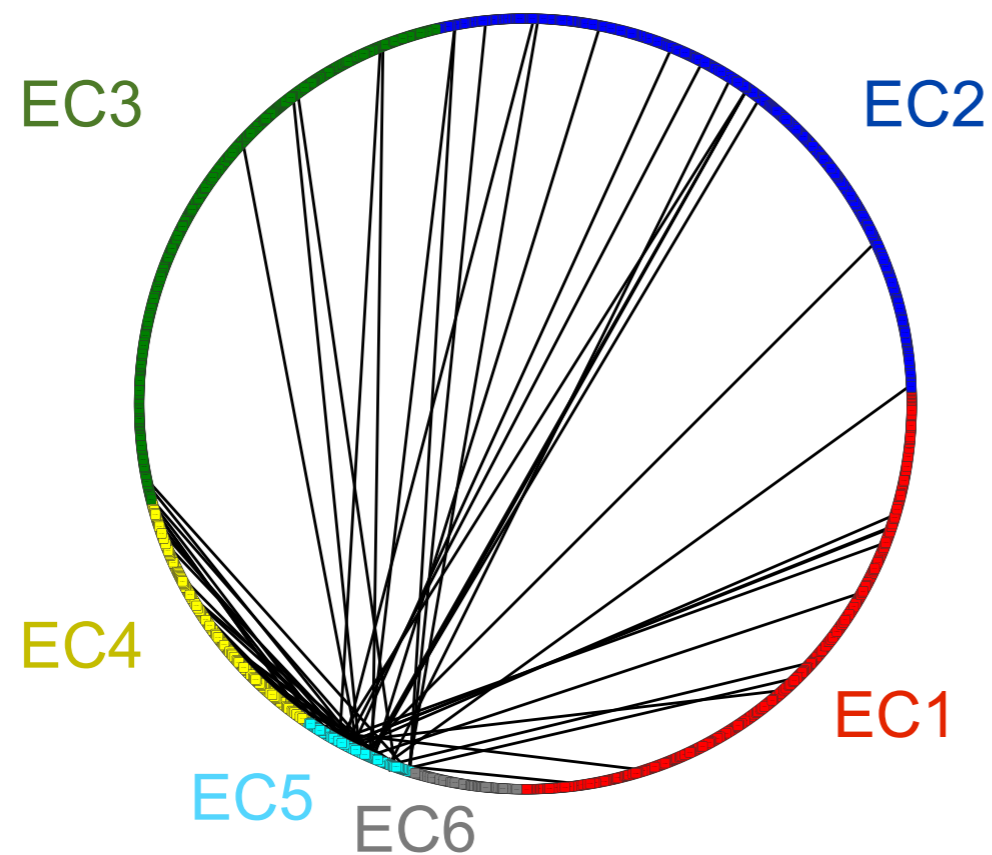

|      |     |     |     |    |     |            |
|------|-----|-----|-----|----|-----|------------|
| 1014 | 37  | 23  | 27  | 16 | 15  | 1          |
| 44   | 934 | 91  | 78  | 33 | 16  | 2          |
| 21   | 79  | 928 | 36  | 19 | 5   | 3          |
| 16   | 38  | 17  | 191 | 32 | 5   | 4          |
| 10   | 19  | 10  | 33  | 52 | 0   | 5          |
| 13   | 14  | 7   | 7   | 1  | 213 | 6          |
| 1    | 2   | 3   | 4   | 5  | 6   | E.C. Class |

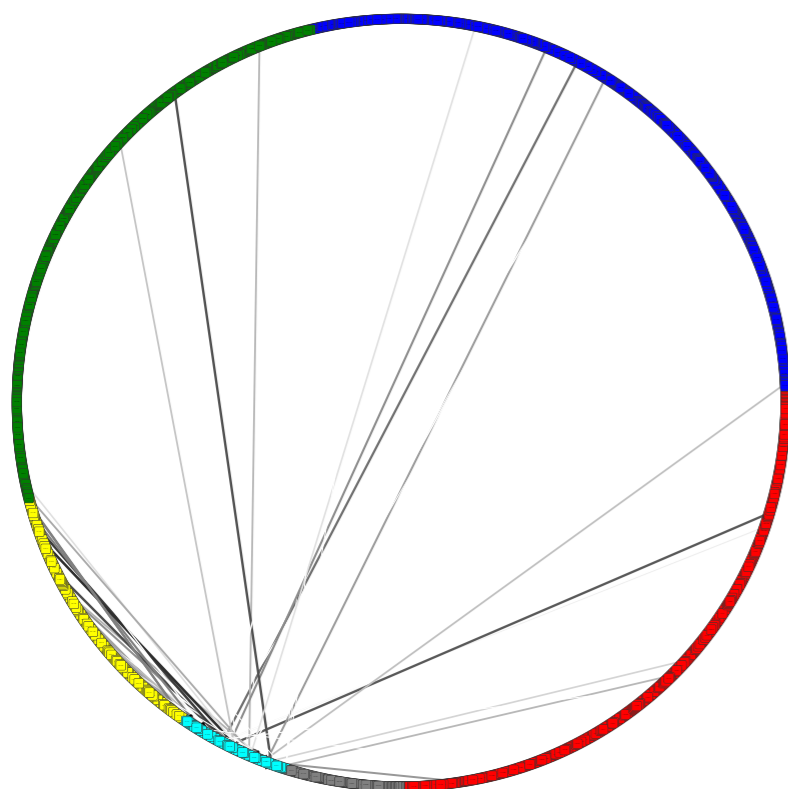

Bond Similarity

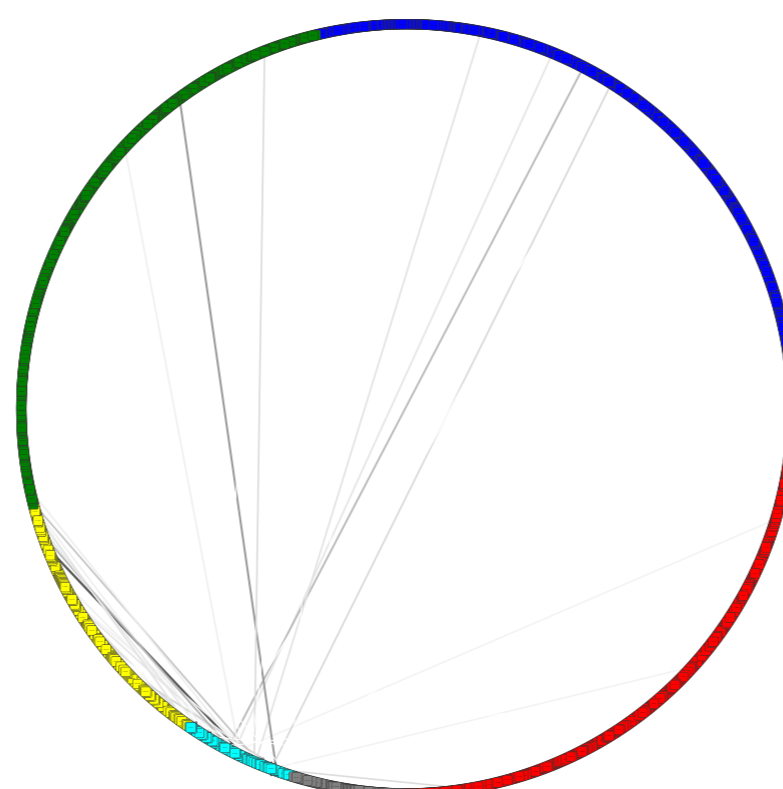

Reaction Centre Similarity

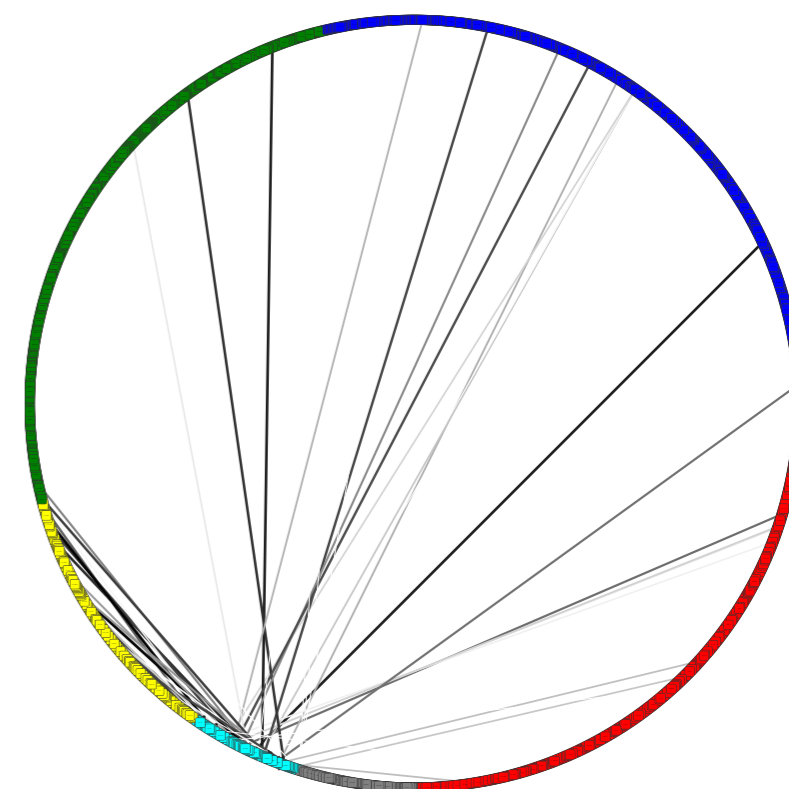

Sub-Structure Similarity

Supplement: Fig. S5 — A summary of changes involving isomerases. All EC numbers in FunTree represented as nodes in a network ordered by EC class. Each change in function associated with a change to/from an isomerase (as highlighted in the EC exchange matrix top right) is shown as an edge in the network (top left). The edges in the networks in the bottom row show the bond change, reaction centre, and sub-structure similarities, respectively, coloured using a grey scale where white is zero similarity and black is exactly the same. [file mmc5.pdf]

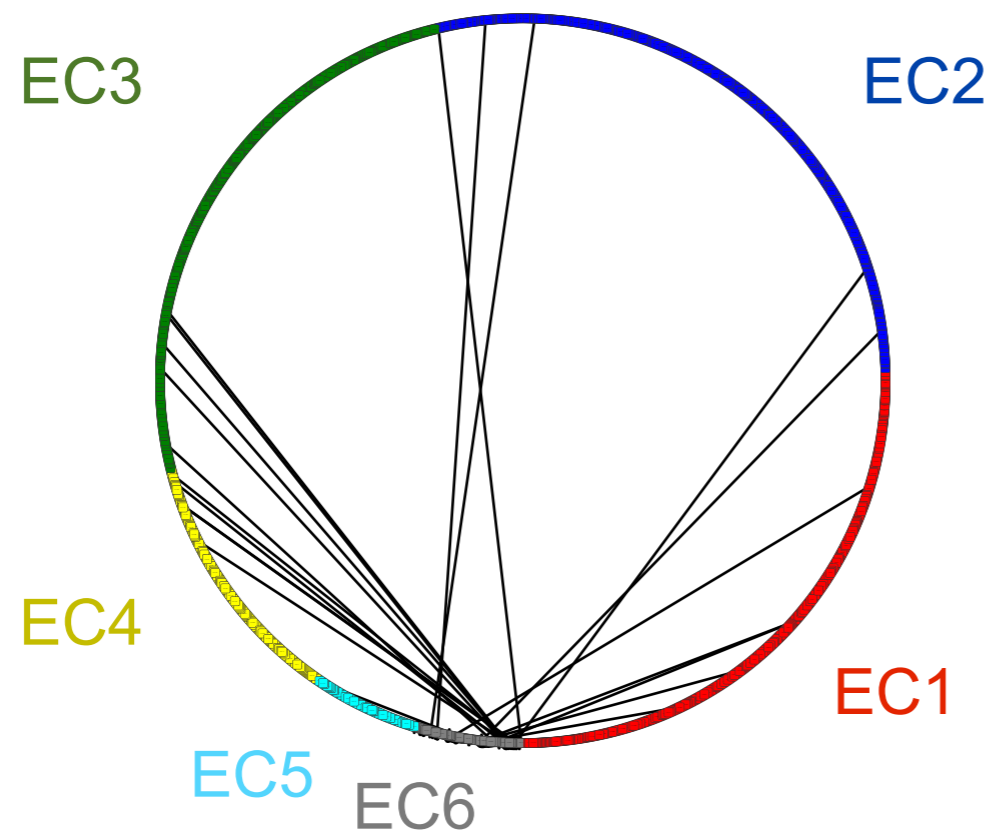

|      |     |     |     |    |     |            |
|------|-----|-----|-----|----|-----|------------|
| 1014 | 37  | 23  | 27  | 16 | 15  | 1          |
| 44   | 934 | 91  | 78  | 33 | 16  | 2          |
| 21   | 79  | 928 | 36  | 19 | 5   | 3          |
| 16   | 38  | 17  | 191 | 32 | 5   | 4          |
| 10   | 19  | 10  | 33  | 52 | 0   | 5          |
| 13   | 14  | 7   | 7   | 1  | 213 | 6          |
| 1    | 2   | 3   | 4   | 5  | 6   | E.C. Class |

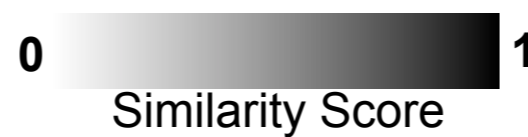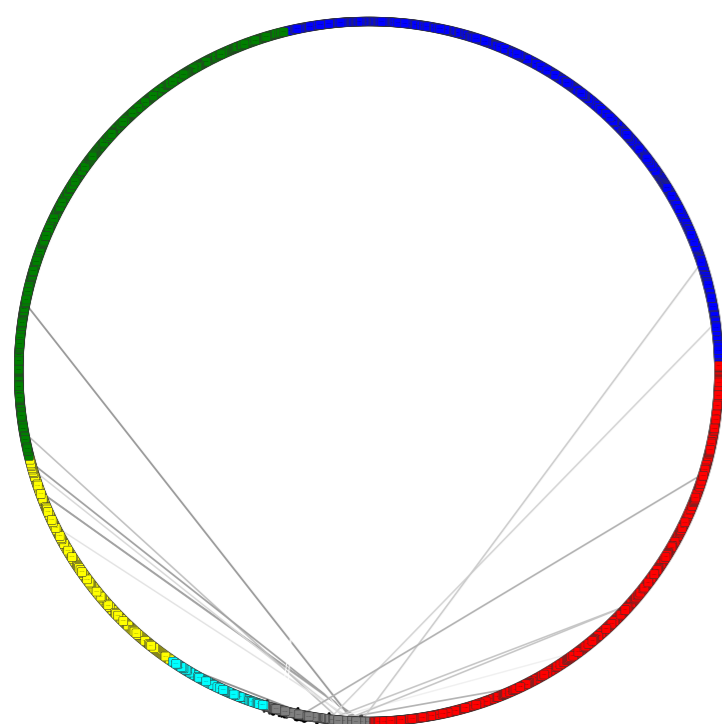

Bond Similarity

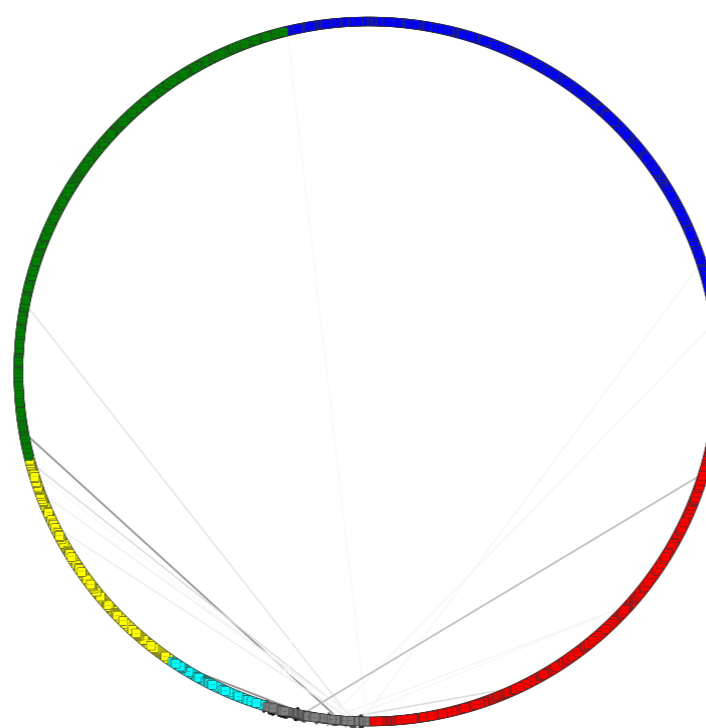

Reaction Centre Similarity

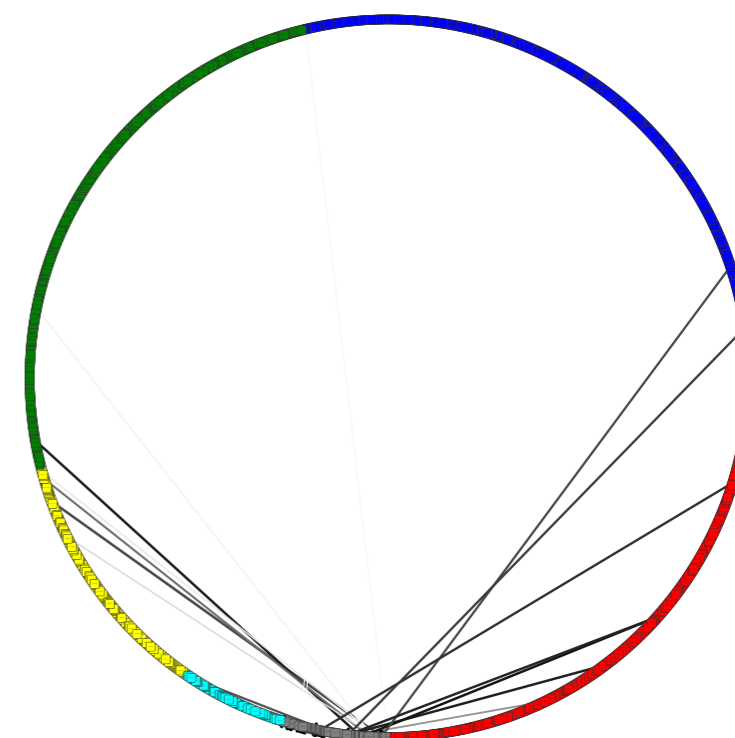

Sub-Structure Similarity

Supplement: Fig. S6 — A summary of changes involving ligases. All EC numbers in FunTree represented as nodes in a network ordered by EC class. Each change in function associated with a change to/from an ligase (as highlighted in the EC exchange matrix top right) is shown as an edge in the network (top left). The edges in the networks in the bottom row show the bond change, reaction centre, and sub-structure similarities, respectively, coloured using a grey scale where white is zero similarity and black is exactly the same. [file mmc6.pdf]

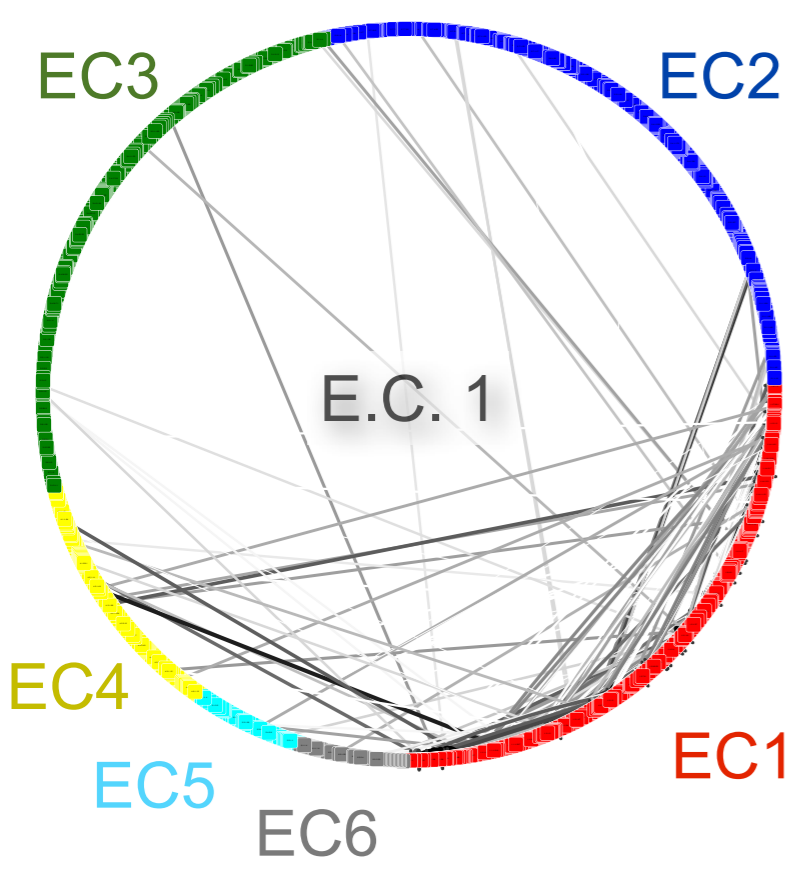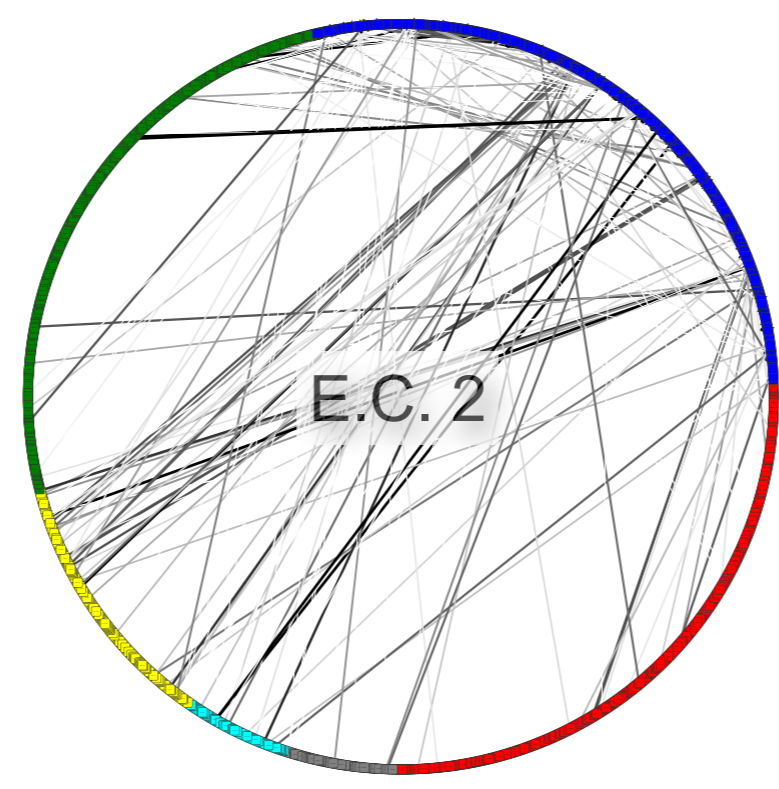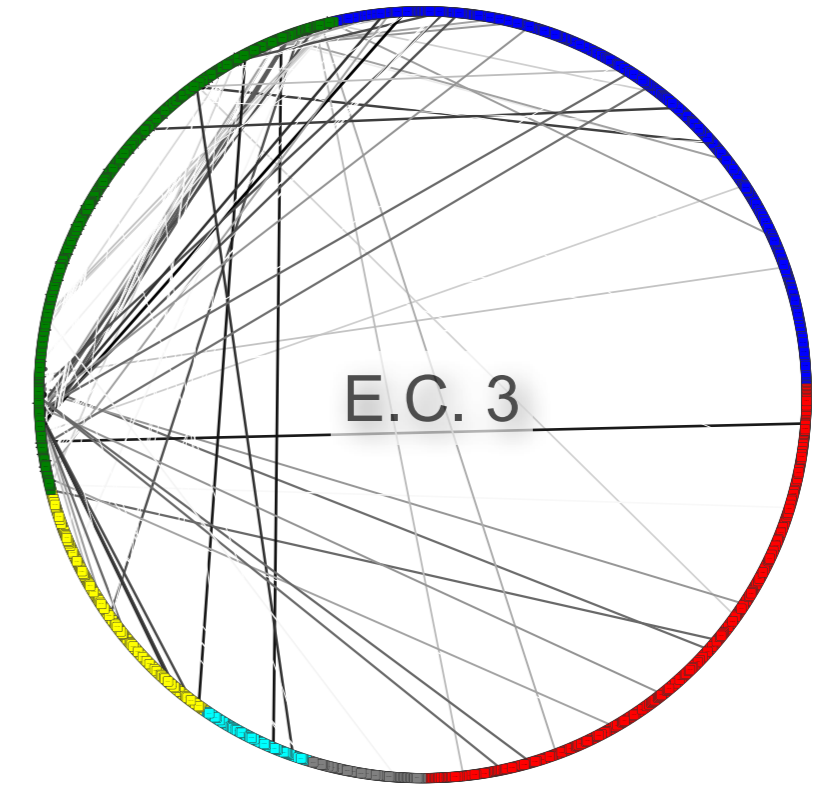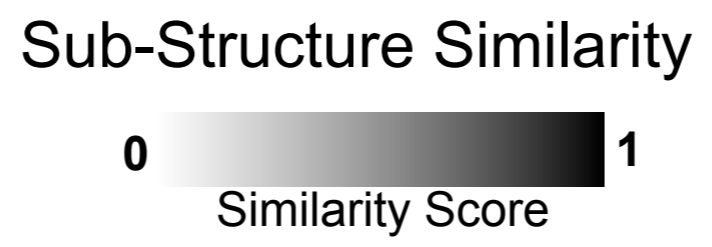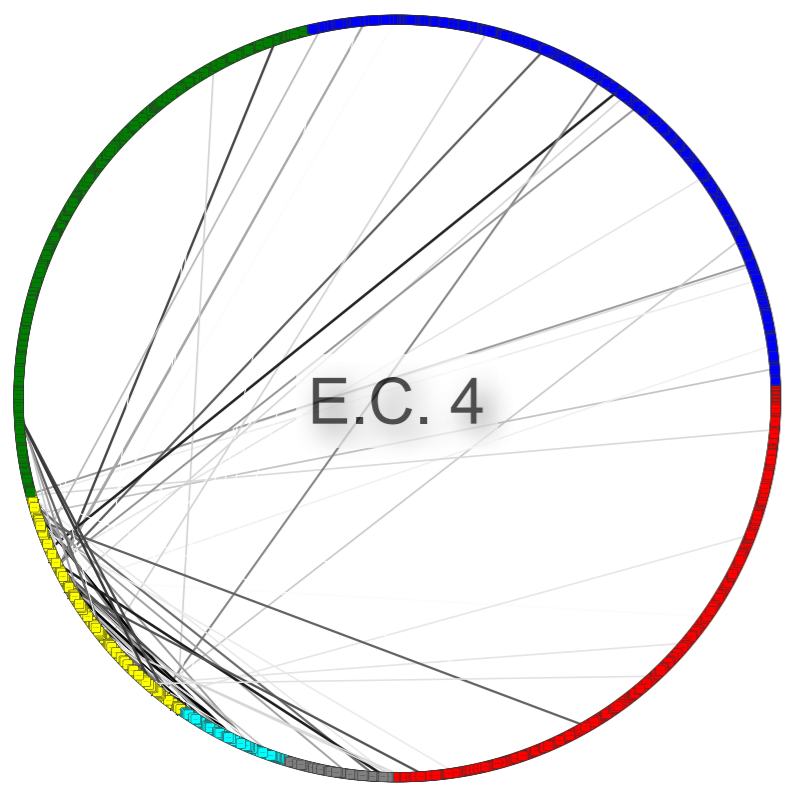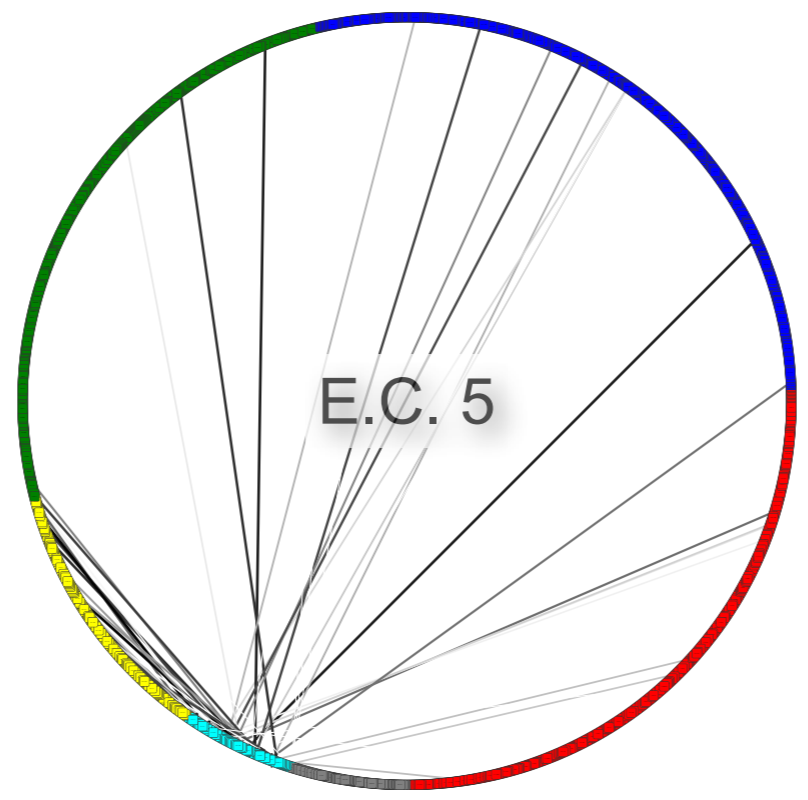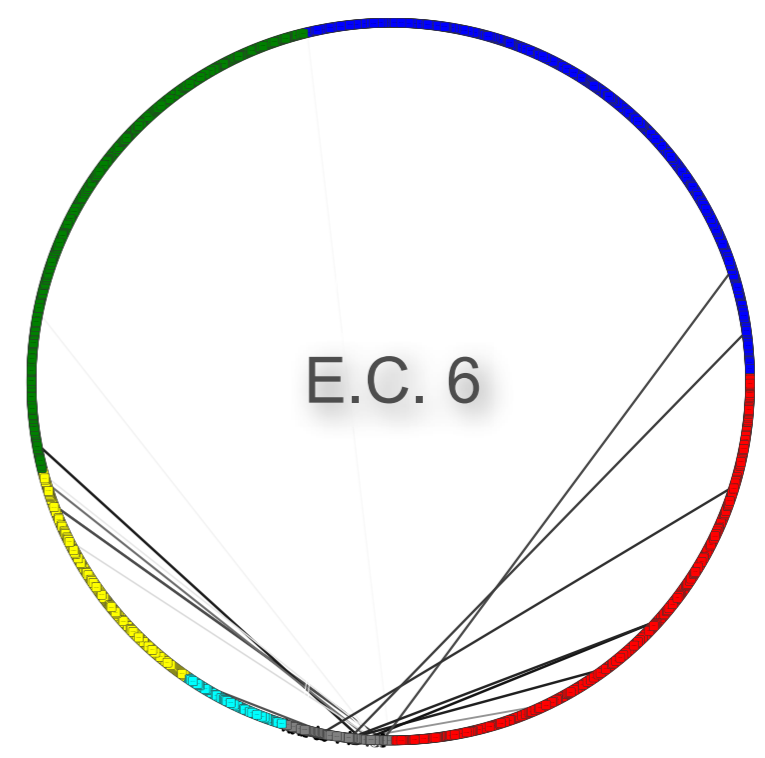

Supplement: Fig. S7 — Comparing bond order similarities by EC class. All EC numbers in FunTree represented as nodes in a network ordered by EC class. Each change in function associated with a change to/from each EC class is shown in each of the networks as an edge. The edge colour shows the reaction centre similarity score using a grey scale where white is zero similarity and black is exactly the same. [file mmc7.pdf]

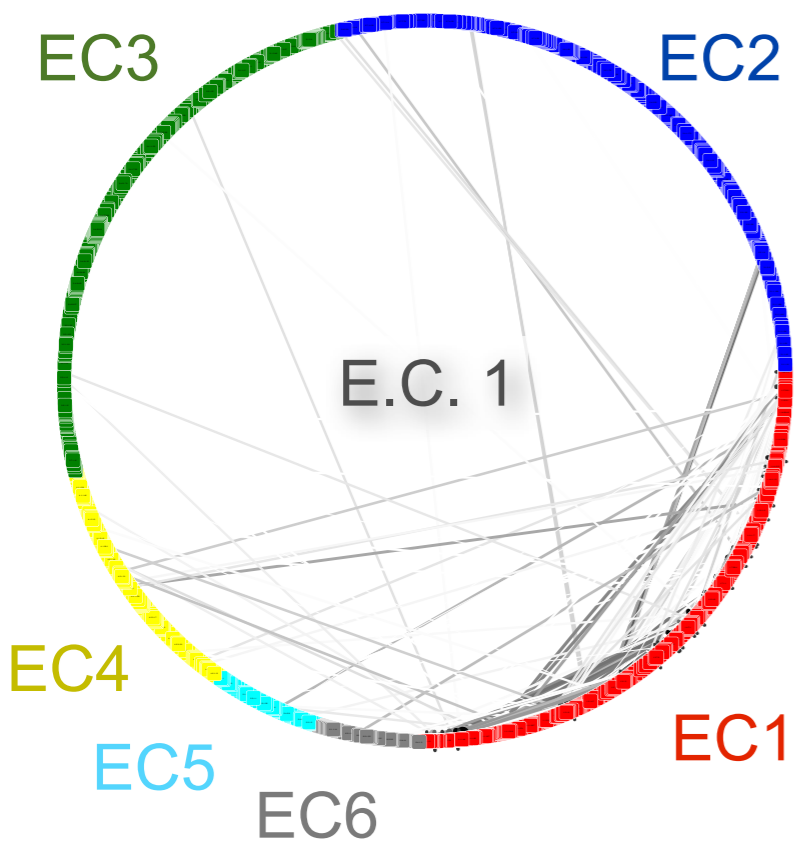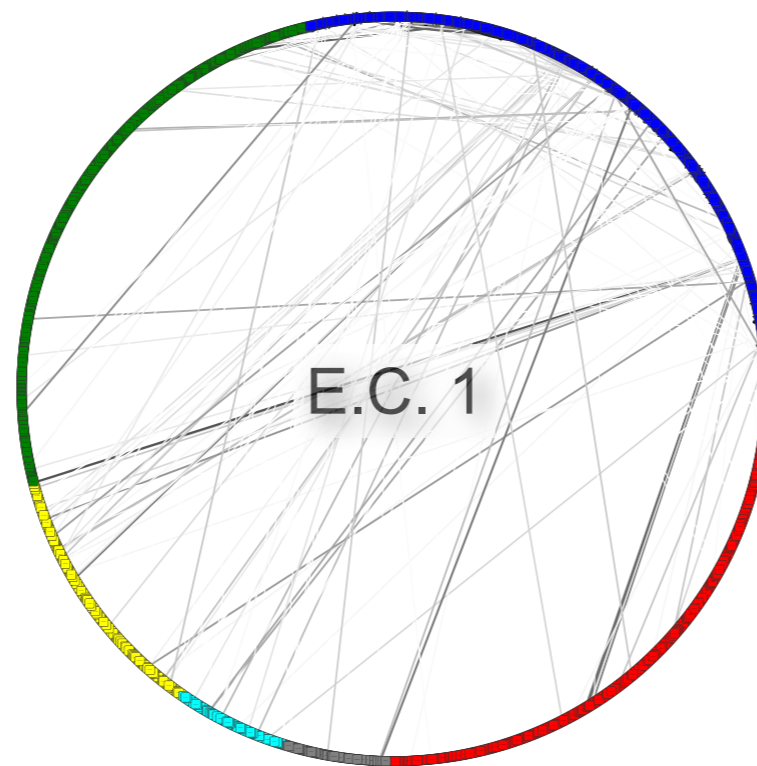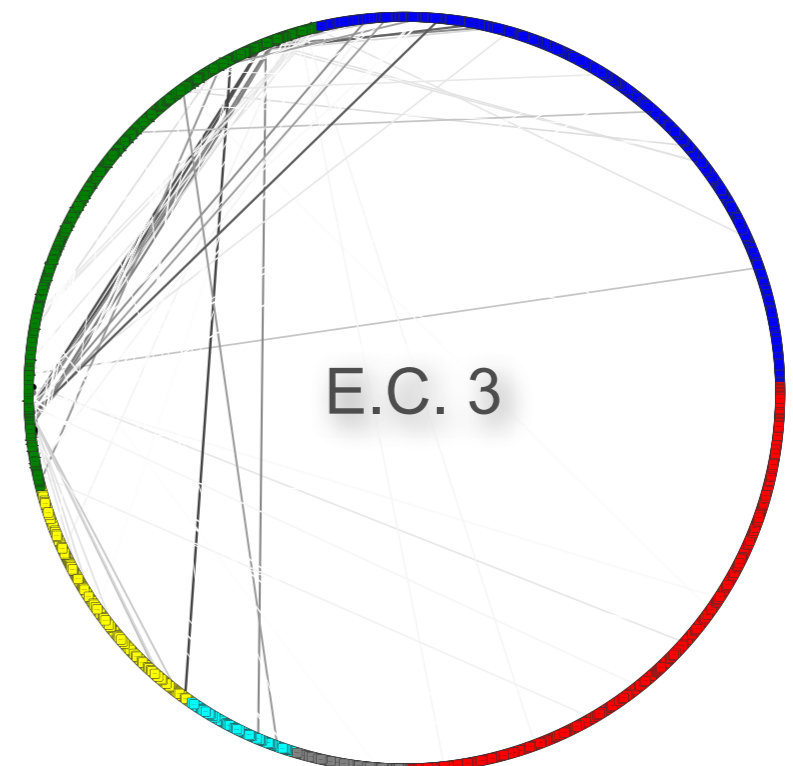

Reaction Centre Similarity

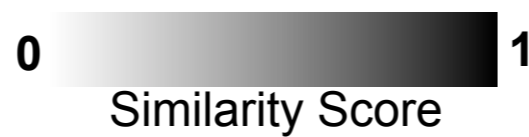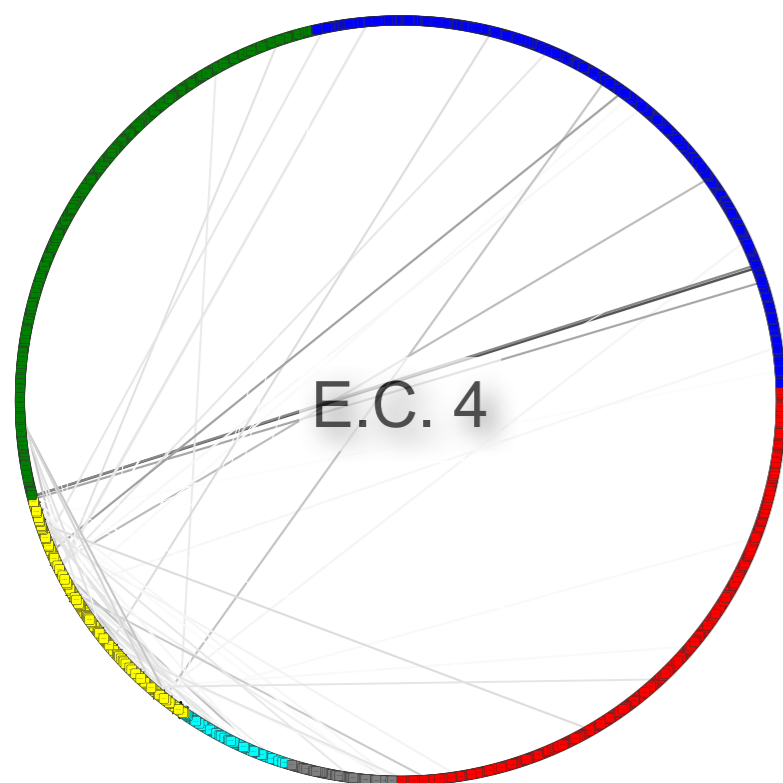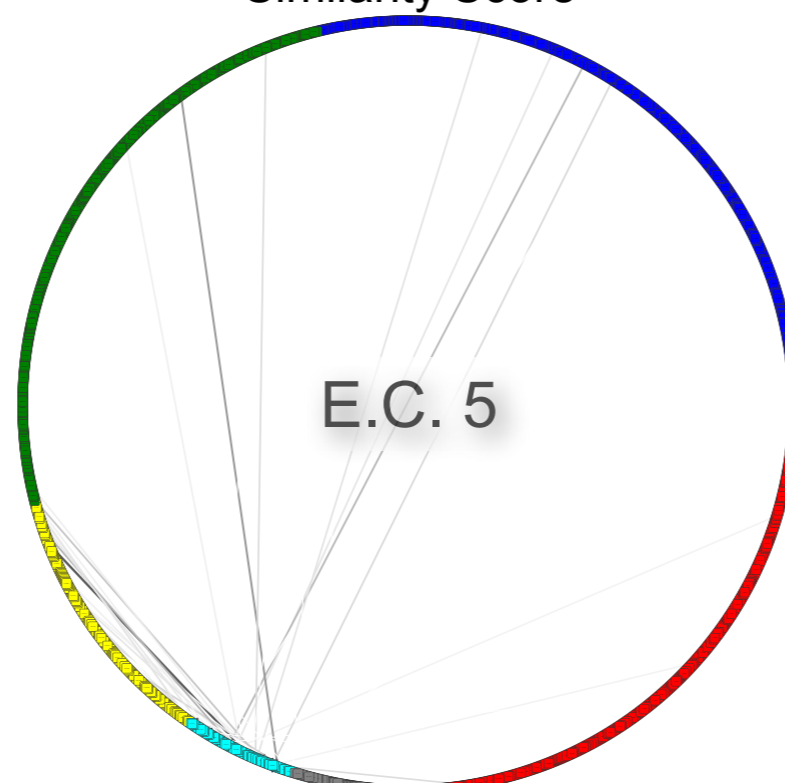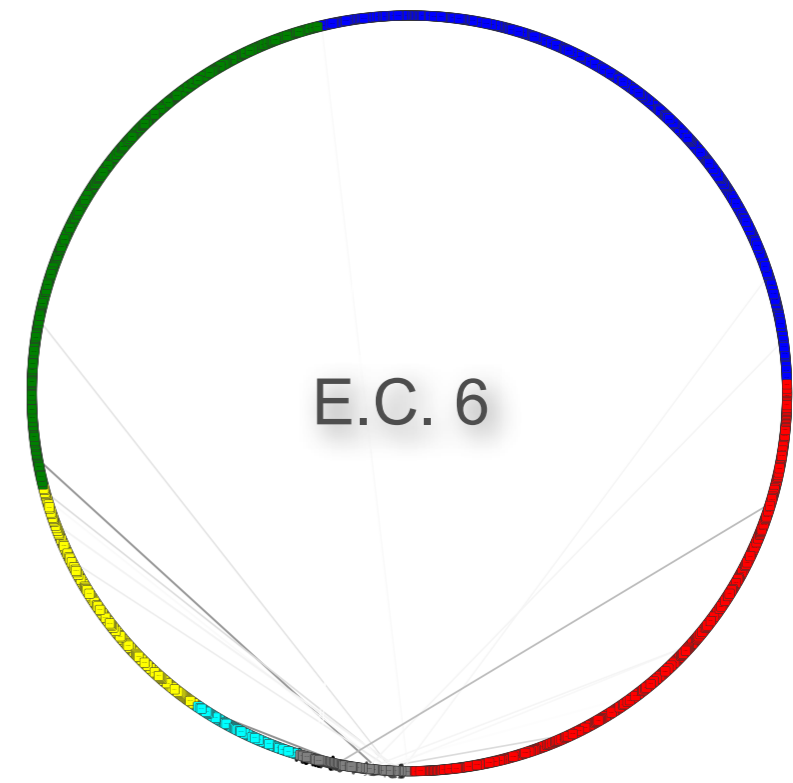

Supplement: Fig. S9 — Comparing sub-structure similarities by EC class. All EC numbers in FunTree represented as nodes in a network ordered by EC class. Each change in function associated with a change to/from each EC class is shown in each of the networks as an edge. The edge colour shows the sub-structure similarity score using a grey scale where white is zero similarity and black is exactly the same. [file mmc9.pdf]

# Bond Conservation

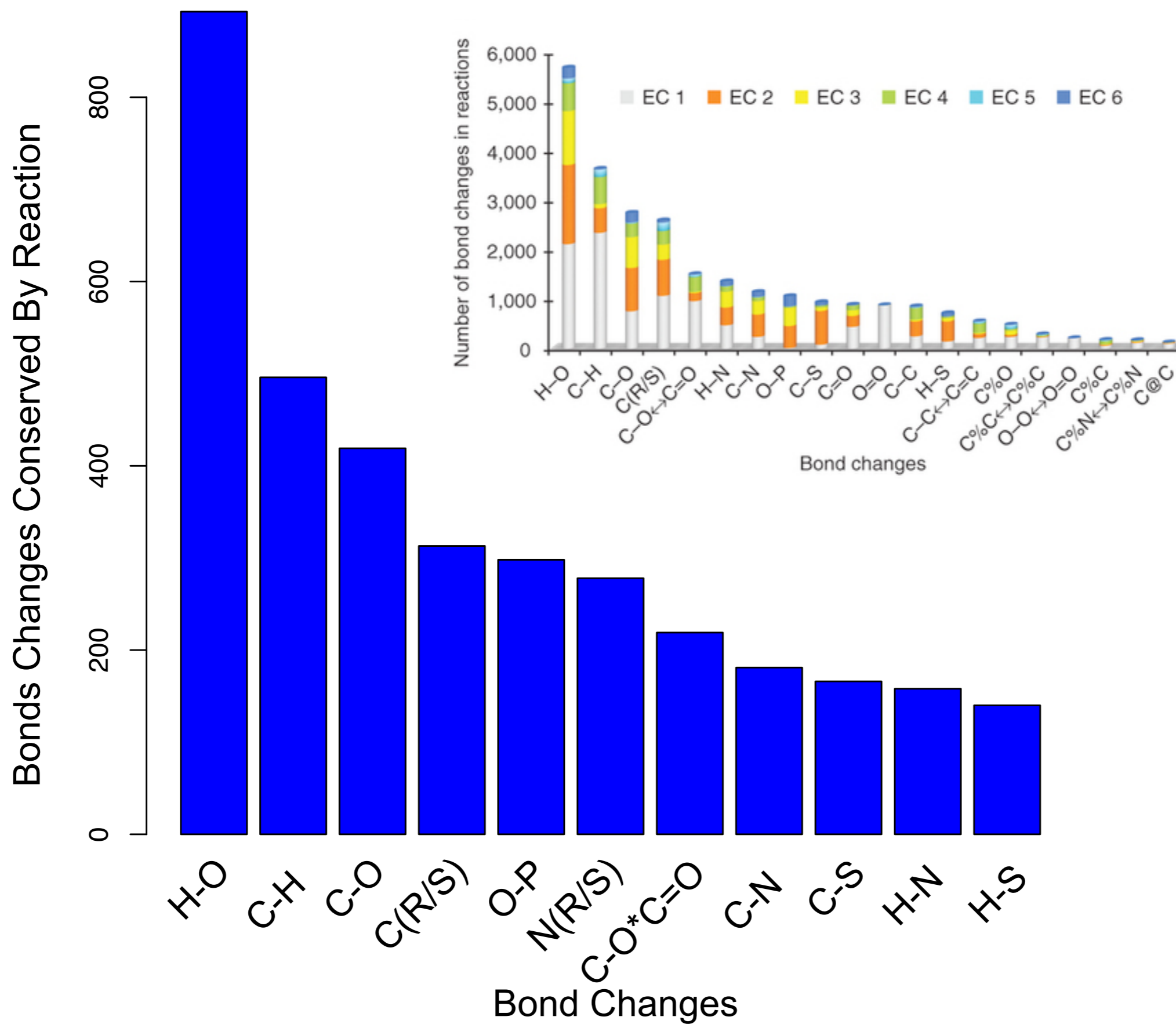

Supplement: Fig. S12 — Conservation of bond types between changes in function. The top 10 cumulative counts of bond types that remain the same between changes in function across all the changes observed in FunTree. The inset shows the distribution of the top 20 bond changes across all known reactions. [file mmc12.pdf]

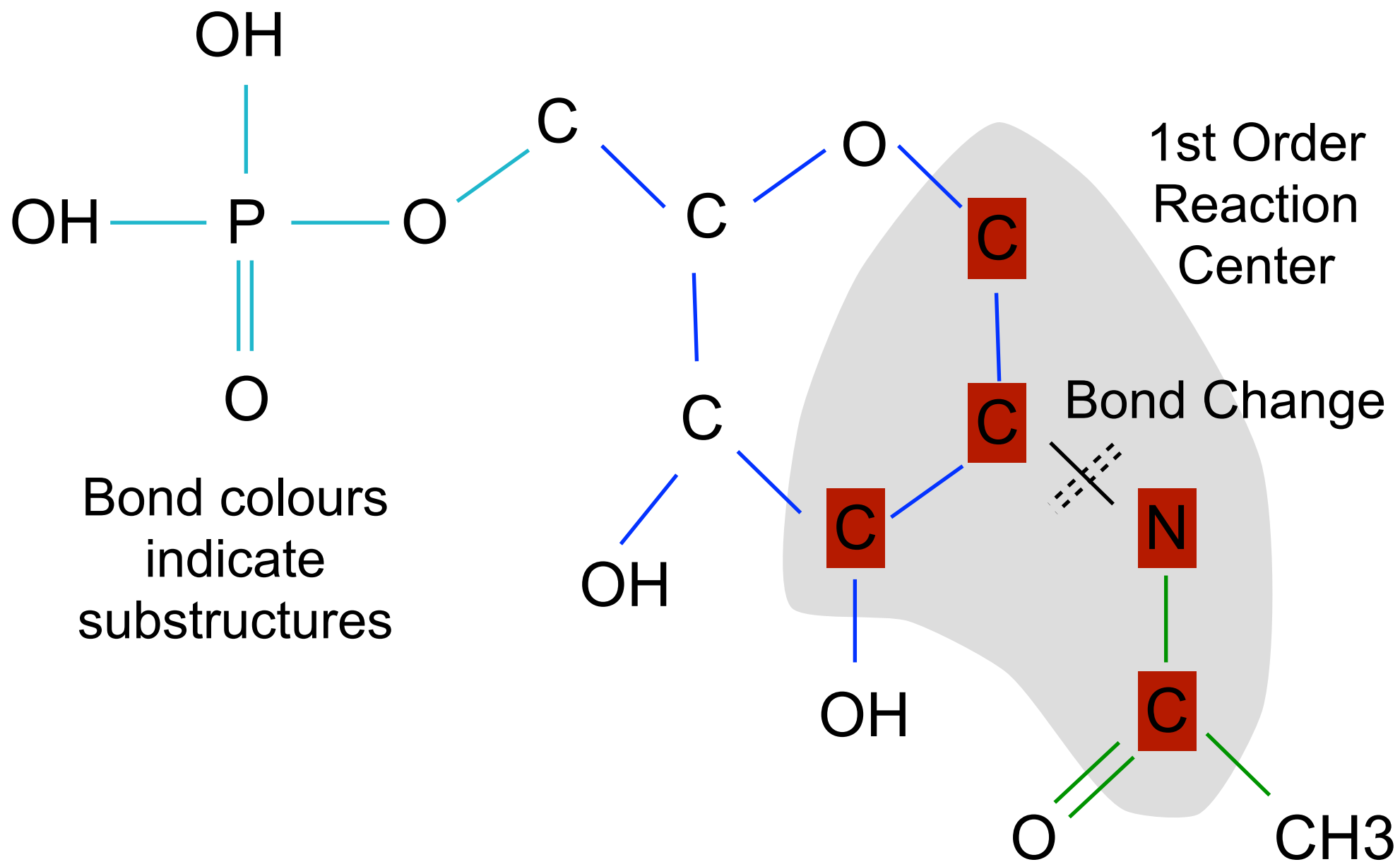

Supplement: Fig. S21 — Flowchart of catalytic residue similarity analysis. The steps taken in calculating catalytic residue similarity values for all pairs of functional families in each superfamily analysed. [file mmc14.pdf]
