# Supplementary material for: Large-Scale Analysis Exploring Evolution of Catalytic Machineries and Mechanisms in Enzyme Superfamilies
Source: J Mol Biol. 2016 Jan 29;428(2Part A):253–67. doi: 10.1016/j.jmb.2015.11.010 (PMC4751976; doi:10.1016/j.jmb.2015.11.010)
Supplement: Fig. S8 — Comparing reaction centre similarities by EC class. All EC numbers in FunTree represented as nodes in a network ordered by EC class. Each change in function associated with a change to/from each EC class is shown in each of the networks as an edge. The edge colour shows the reaction centre similarity score using a grey scale where white is zero similarity and black is exactly the same. [file mmc8.pdf]

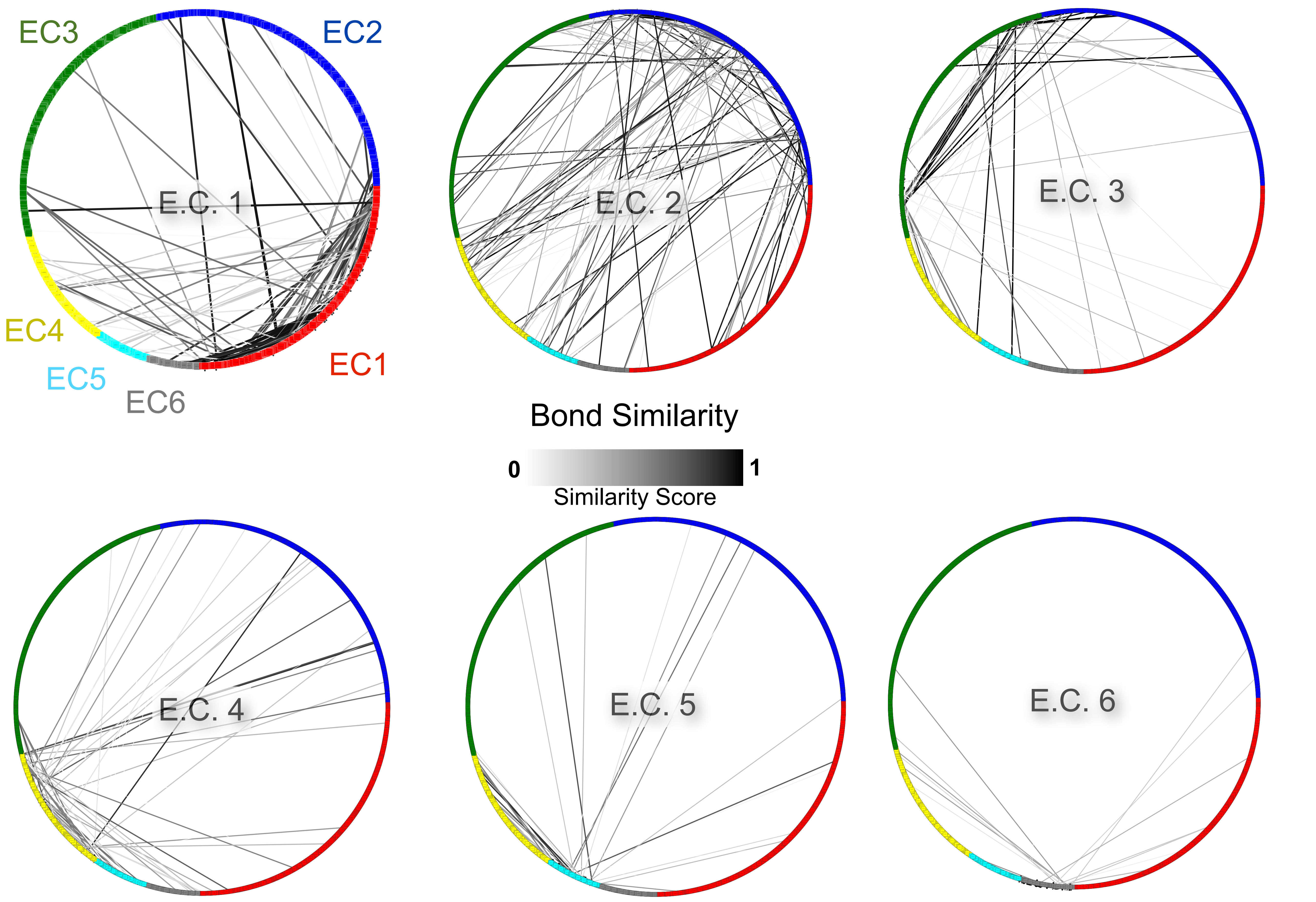
EC3
EC2
E.C. 1
EC4
EC1
EC5
EC6
E.C. 2
E.C. 3
Bond Similarity
0
1
Similarity Score
E.C. 4
E.C. 5
E.C. 6
