# Supplementary material for: Large-Scale Analysis Exploring Evolution of Catalytic Machineries and Mechanisms in Enzyme Superfamilies
Source: J Mol Biol. 2016 Jan 29;428(2Part A):253–67. doi: 10.1016/j.jmb.2015.11.010 (PMC4751976; doi:10.1016/j.jmb.2015.11.010)
Supplement: Fig. S10 — Summary of Vanillyl-Alcohol Oxidase superfamily (CATH ID 1.10.45.10). (a) Overall reaction diagrams with sub-structures identified by SMSD coloured for the two reactions found in the superfamily: vanillyl-alcohol oxidase (EC 1.1.3.38) and 4-methylphenol dehydrogenase (EC 1.17.99.2). (b) A summary table of bond changes for each reaction. [file mmc10.pdf]

A

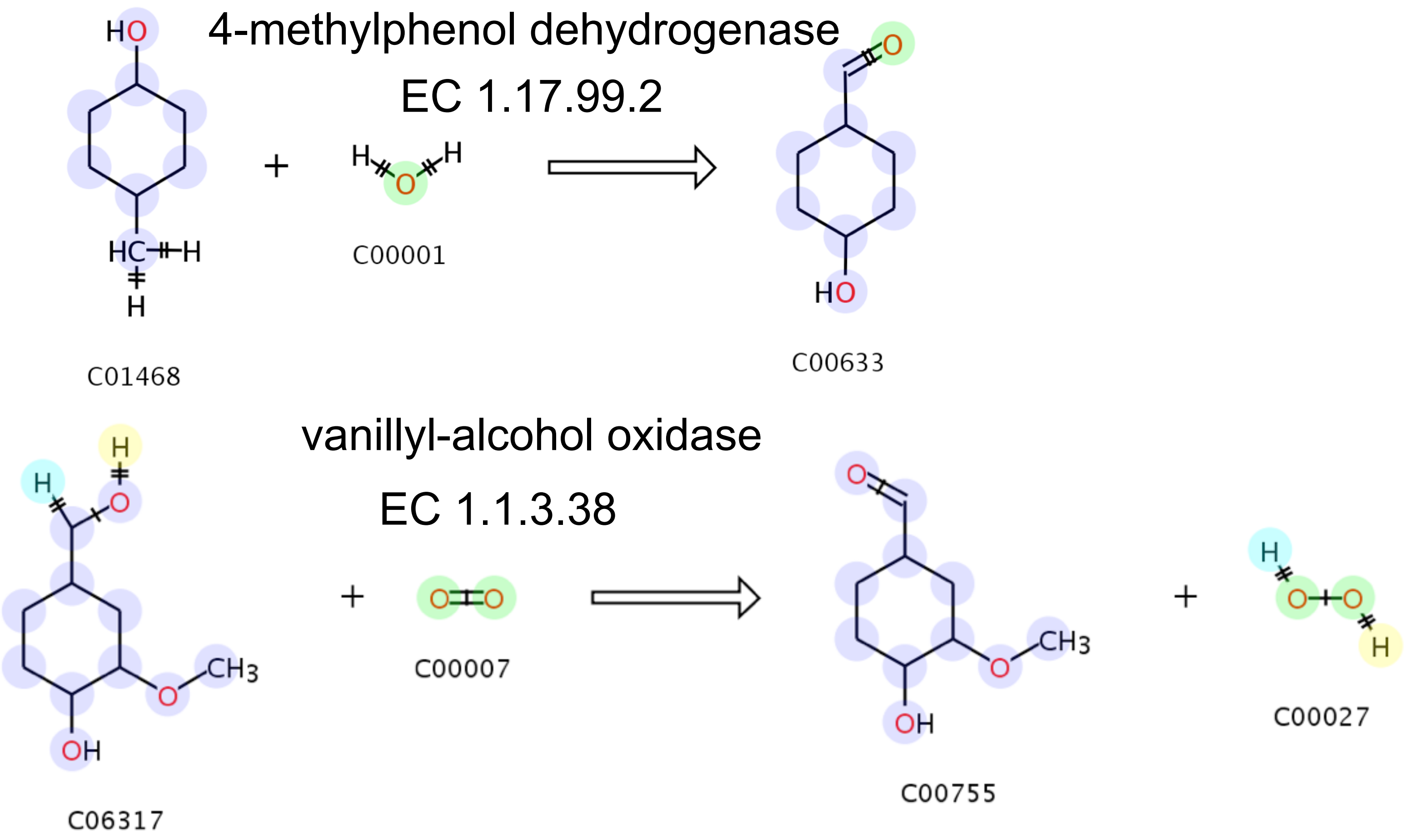

B

Bond Changes

EC 1.17.99.2

Formed/Cleaved (⧺)		Bond Order Changed (⧧)		Stereo Changes (◁)	
C-O	1			C(R/S)	1

EC 1.1.3.38

Formed/Cleaved (⧺)		Bond Order Changed (⧧)		Stereo Changes (◁)
H-O	3	O-O<>O=O	1	
C-H	1	C-O<>C=O	1	

EC-Blast Comparison of Bond Changes = 0; Similarity of reactants = 0.6
