# Supplementary material for: Large-Scale Analysis Exploring Evolution of Catalytic Machineries and Mechanisms in Enzyme Superfamilies
Source: J Mol Biol. 2016 Jan 29;428(2Part A):253–67. doi: 10.1016/j.jmb.2015.11.010 (PMC4751976; doi:10.1016/j.jmb.2015.11.010)
Supplement: Fig. S11 — Summary of Amidase Signature enzyme superfamily (CATH ID 3.90.1300.10) reactions. (a) Overall reaction diagrams with sub-structures identified by SMSD coloured for two of the reactions found in the superfamily: amidase (EC 3.5.1.4) and 6-aminohexanoate-cyclic-dimer hydrolase (EC 3.5.2.12). (b) A summary table of bond changes for each reaction. (c) A summary table of conservation of catalytic residues in the sequences of the other enzymes in the superfamily, with a tick indication conservation and a cross indication that it is not conserved. [file mmc11.pdf]

A

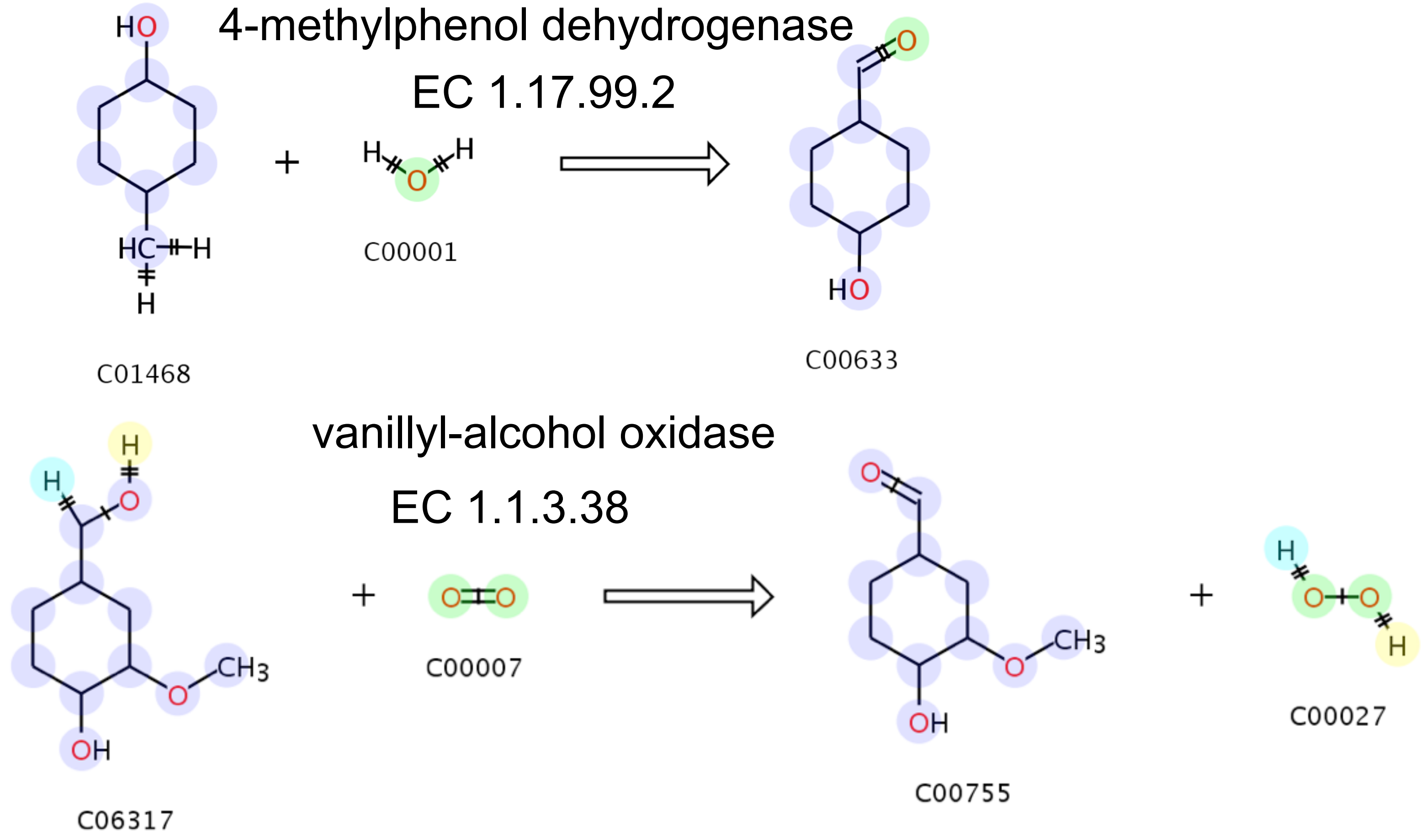

B

Bond Changes

EC 1.17.99.2

Formed/Cleaved		Bond Order Changed		Stereo Changes	
C-O	1			C(R/S)	1

EC 1.1.3.38

Formed/Cleaved		Bond Order Changed		Stereo Changes	
H-O	3	O-O<>O=O	1		
C-H	1	C-O<>C=O	1		

EC-Blast Comparison of Bond Changes = 0; Similarity of reactants = 0.6
