# Supplementary material for: Large-Scale Analysis Exploring Evolution of Catalytic Machineries and Mechanisms in Enzyme Superfamilies
Source: J Mol Biol. 2016 Jan 29;428(2Part A):253–67. doi: 10.1016/j.jmb.2015.11.010 (PMC4751976; doi:10.1016/j.jmb.2015.11.010)
Supplement: Fig. S19 — Reaction centre. A first-order reaction centre is defined as the atoms involved in the bond change and those atoms directly connected to them. The reaction centre is highlighted in grey and the atoms that make up the reaction centre are highlighted in red. [file mmc13.pdf]

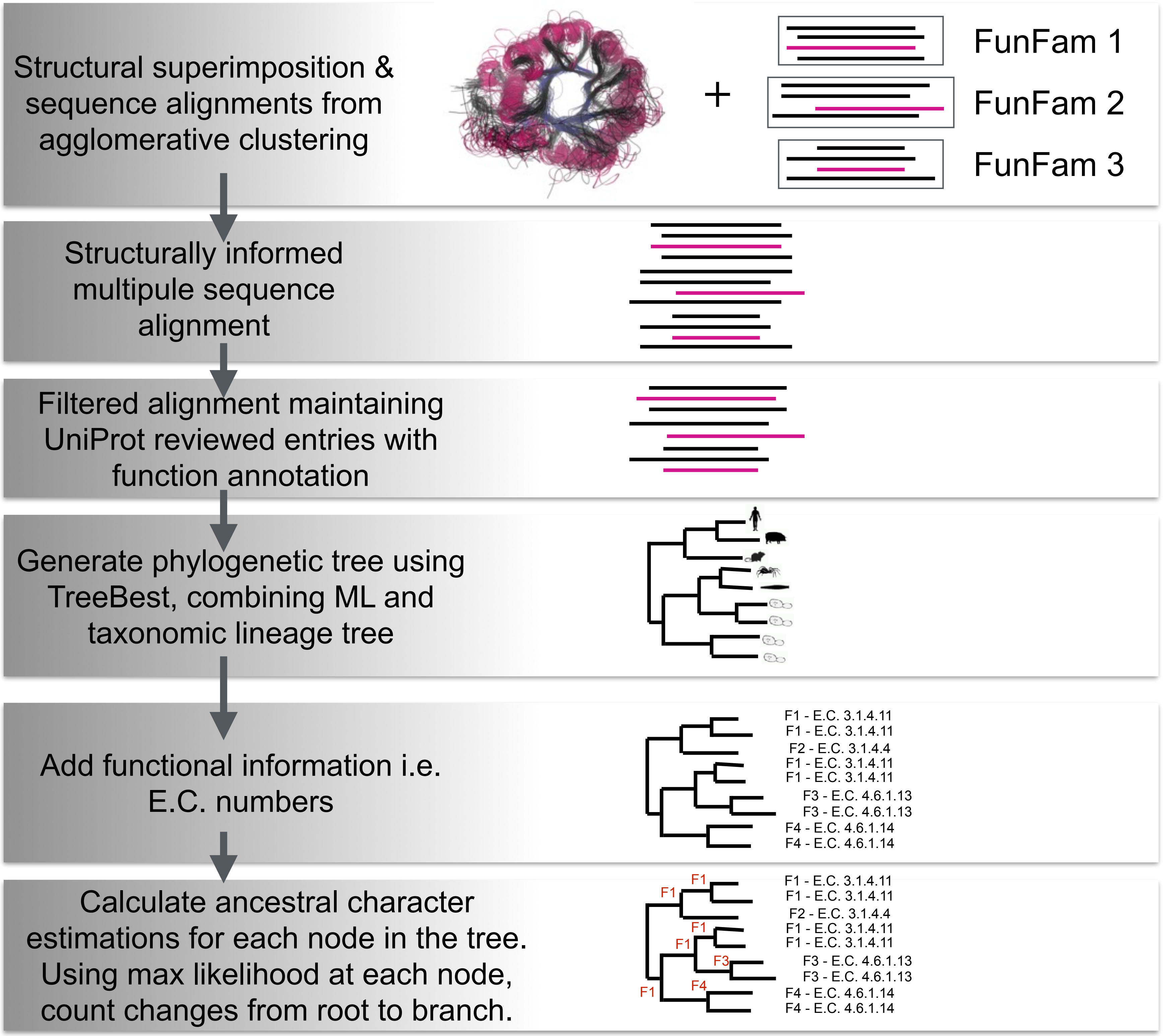
Structural superimposition & sequence alignments from agglomerative clustering
+
FunFam 1
FunFam 2
FunFam 3
Structurally informed multipule sequence alignment
Filtered alignment maintaining UniProt reviewed entries with function annotation
Generate phylogenetic tree using TreeBest, combining ML and taxonomic lineage tree
Add functional information i.e. E.C. numbers
F1 - E.C. 3.1.4.11
F1 - E.C. 3.1.4.11
F2 - E.C. 3.1.4.4
F1 - E.C. 3.1.4.11
F1 - E.C. 3.1.4.11
F3 - E.C. 4.6.1.13
F3 - E.C. 4.6.1.13
F4 - E.C. 4.6.1.14
F4 - E.C. 4.6.1.14
Calculate ancestral character estimations for each node in the tree. Using max likelihood at each node, count changes from root to branch.
F1
F1
F1
F1
F3
F4
F1
F1 - E.C. 3.1.4.11
F1 - E.C. 3.1.4.11
F2 - E.C. 3.1.4.4
F1 - E.C. 3.1.4.11
F1 - E.C. 3.1.4.11
F3 - E.C. 4.6.1.13
F3 - E.C. 4.6.1.13
F4 - E.C. 4.6.1.14
F4 - E.C. 4.6.1.14
